# A DNA-Local, Constraint-Aware Dual-Head Transformer for Pseudorandom Stream Generation

**DOI:** 10.3390/e28060694

**Published:** 2026-06-16

**Authors:** Alev Kaya, İbrahim Türkoğlu

**Affiliations:** 1 Software Engineering Graduate Program, Graduate School of Natural and Applied Sciences, Firat University, 23119 Elazig, Türkiye; 2 Department of Software Engineering, Faculty of Technology, Firat University, 23119 Elazig, Türkiye; iturkoglu@firat.edu.tr

**Keywords:** DNA-local PRNG, constraint-aware generation, dual-head Transformer, adaptive rule selection, genomic sequence modeling, randomness evaluation

## Abstract

Pseudorandom number generators (PRNGs) used in deoxyribonucleic acid (DNA)-oriented computational workflows often generate outputs in the bit domain and then map them to DNA symbols. This indirect strategy may treat DNA-specific constraints, including GC balance, homopolymer limits, and short-range sequence dependencies, as separate from generation. This study proposes a constraint-aware, dual-head decoder-only Transformer framework for DNA-local PRNG generation directly in the adenine/cytosine/guanine/thymine (A/C/G/T) alphabet. The model generates the next DNA base and derives the bitstream through dynamic selection among eight equivalent DNA-to-bit coding rules. The framework was evaluated under R1 based on real genomic data, R1-ext as independent validation, R2 based on synthetic data, and R3 without training or reference data. For each setting, 10 independent runs were performed, each producing a 500,000-base DNA sequence and a 1,000,000-bit stream. Bit-level evaluation used NIST SP 800-22, SP 800-90B-inspired min-entropy/health indicators, and ENT, while DNA-level evaluation used GC balance, homopolymer control, and symbolic structural metrics. The reported NIST tests satisfied the acceptance criterion, t-tuple min-entropy lower bounds ranged from 0.9955 to 0.9964 bit/bit, and core DNA-compatibility constraints were preserved. Multi-stream and exact-match k-mer leakage analyses indicated no systematic bit-level dependence or direct long-fragment copying. Overall, the framework supports reproducible DNA-local PRNG generation and multilayer validation.

## 1. Introduction

Randomness is a fundamental component of modern computational systems used in data processing, modeling, simulation, security, and privacy-oriented applications [1,2]. This requirement becomes more specific in deoxyribonucleic acid (DNA)-based computing and biomolecular workflows. In processes such as DNA synthesis, sequencing, indexing, error modeling, data storage, and biomolecular design, it is not sufficient for sequences to appear statistically random. They must also satisfy structural and process-related constraints of DNA [3,4]. In particular, guanine/cytosine (GC) balance, homopolymer length, short-range dependencies, local motif enrichment, and k-mer distribution can directly affect the synthesizability, sequenceability, and downstream analytical reliability of DNA-based outputs [3,4,5,6].

Many pseudorandom number generators (PRNGs) used in DNA-oriented applications first generate outputs in the bit domain and then convert these outputs into the adenine/C/G/thymine (A/C/G/T) base alphabet [5,6]. Although this bit-first strategy is practical and widely used, it does not treat DNA-specific structural properties as an intrinsic part of the generation process. A bitstream that appears statistically acceptable at the bit level may produce GC imbalance, long homopolymer runs, or local motif accumulation after being mapped to the DNA alphabet. Moreover, the transition to DNA is often implemented as a fixed mapping step after generation. This makes it difficult to manage DNA-level structural compatibility and bit-level statistical randomness within the same generation cycle. The use of a single fixed DNA-to-bit mapping rule may also introduce secondary regularities in the bitstream. Therefore, a PRNG core designed for DNA-based workflows should generate directly in the DNA alphabet, account for DNA-specific constraints during generation, and derive the bitstream in a traceable manner.

In recent years, learning-based PRNG designs, hybrid systems involving DNA coding, and Transformer-based generative models have attracted increasing attention in this field [7,8,9,10,11,12,13]. However, many existing studies still rely on bit-domain generation or treat the transition to DNA representation as a post hoc coding layer [5,6]. Data-set dependence also remains an important limitation in learning-based methods. When models are trained on large data sets or on data sets with specific distributional properties, it is not always clear whether the observed output quality originates from the model architecture or from patterns transferred from the training data. This issue is more pronounced for DNA sequences, because genomic sequences contain natural regularities at the levels of species, genomic region, motif composition, and local dependency. Therefore, a DNA-local PRNG approach should be evaluated not only under data-supported conditions, but also under conditions in which no training data are used.

These trends in the current literature limit the joint treatment of DNA-specific structural compatibility and bit-level statistical randomness within the same generation core. In addition, the successful performance of a model trained only on data does not demonstrate that the method has a data-independent generation capacity. There is therefore a need for an integrated PRNG design that operates directly in the DNA alphabet, manages DNA-specific constraints during generation, derives the bitstream through dynamic rule selection, and compares data-supported and data-independent conditions under the same architectural core.

In response to this need, this study proposes a DNA-local, constraint-aware, dual-head Transformer-based PRNG core. The proposed structure generates outputs directly in the A/C/G/T alphabet and simultaneously derives the corresponding bitstream. The first output head produces the probability distribution for the next DNA base, whereas the second output head models the selection behavior among eight equivalent DNA-to-bit coding rules. During generation, the GC ratio, maximum homopolymer length, short-range dependency, and local n-gram behavior are regulated at the sampling level. Thus, DNA sequence generation, DNA-to-bit conversion, and structural constraint management are not treated as disconnected sequential steps. Instead, they are handled as interacting components of the same generation pipeline.

The aim of this study is not to train a large-scale language model or to reproduce a specific genomic data set. Rather, the aim is to develop a computationally traceable PRNG core that can operate with lightweight calibration and generate structurally compatible outputs for DNA-based production workflows. For this reason, a limited sub-corpus and a single-epoch training scheme are used in data-supported regimes, while generation-time constraints guide model outputs according to DNA-specific structural criteria. More importantly, this study also defines a reference-free generation regime in which no training data are used. This no-reference/no-training regime provides a critical control condition for evaluating whether the proposed core depends only on learned genomic patterns.

The proposed method is examined under three main regimes. The R1 regime represents the data-supported setting trained on real genomic DNA [14]. The R2 regime represents the controlled setting trained on approximately uniform synthetic DNA [15]. The R3 regime represents the reference-free setting that operates without any training data. This three-regime design aims to separate the effect of data from the architectural structure and the constraint-aware sampling mechanism. In addition, external validation on an independent genome is performed to reduce dependence on a single real genomic source. This additional analysis is used to assess more clearly whether the method behaves as a data-dependent generator tied to a specific genomic source.

This study does not propose a cryptosystem and does not claim to provide a formal proof of cryptographic security. Accordingly, the evaluation is not based on an attack model, key security, or provable security claims. Instead, it focuses on statistical randomness, DNA-specific structural compatibility, data dependence, generation reproducibility, and consistency across regimes. Passing National Institute of Standards and Technology (NIST)-based tests alone should not be interpreted as proof of cryptographic security [16,17,18,19]. Nevertheless, when tests such as NIST Special Publication (SP) 800-22, NIST SP 800-90B, and ENT are used together with DNA-specific metrics, they provide a meaningful initial validation layer for assessing the statistical quality of PRNG outputs [16,17,18,19,20,21]. Therefore, this study evaluates bit-level tests jointly with DNA-level measures such as GC balance, homopolymer profile, k-mer distribution, short-range dependency, rule-usage behavior, and multi-stream independence [3,4,5,6]. The main contributions of this study are summarized as follows:

- A dual-head Transformer-based PRNG architecture is proposed for direct output generation in the DNA alphabet.
- GC balance, homopolymer limitation, short-range dependency control, and local motif behavior are treated as components of a constraint-aware sampling mechanism operating during generation, rather than as post-generation corrections.
- Data-supported and data-independent generation conditions are compared under the same architectural core through the R1, R2, and R3 regimes. In particular, the R3 no-reference/no-training regime enables the assessment of data-set dependence and generation behavior without training data.
- The bitstream is derived through dynamic selection among eight equivalent DNA-to-bit coding rules, rather than through a single fixed mapping rule.
- Statistical randomness tests and DNA-specific structural measures are jointly evaluated within an integrated validation protocol.
- Independent genomic validation, summary statistics across independent runs, and additional analyses of component contributions are used to assess reproducibility and consistency across regimes more explicitly.

The findings show that the proposed method can produce acceptable bit-level statistical randomness behavior under different data regimes. They also show that the generated DNA outputs remain traceable with respect to GC balance, homopolymer control, local motif distribution, and short-range dependency. The remainder of this paper is organized as follows. Section 2 summarizes related work and the research gap. Section 3 describes the proposed architecture and generation-time constraint mechanisms. Section 4 presents the experimental design and results. Section 5 discusses the findings, limitations, and security scope. Finally, Section 6 concludes the study and outlines future research directions.

## 2. Related Work and Research Gap

The literature on randomness generation compatible with DNA-based processes can be considered along three main lines within the scope of this study: learning-based PRNG approaches, hybrid systems involving DNA coding or encryption steps, and Transformer-based PRNG studies. This section reviews these research lines together to position the proposed DNA-local and constraint-aware PRNG core within the literature. The evaluation focuses particularly on the output space, whether DNA-specific constraints are handled during generation, the DNA-to-bit conversion strategy, the availability of a reference-free generation setting, and the scope of the validation protocol.

Learning-based PRNG studies have mainly developed along deep learning and reinforcement learning directions. Architectures such as recurrent neural networks (RNNs), long short-term memory (LSTM) networks, convolutional neural networks (CNNs), generative adversarial networks (GANs), and Wasserstein GANs with gradient penalty (WGAN-GP) have been used to generate binary outputs by learning distributional patterns from training data [22,23,24,25,26,27,28]. LSTM-based approaches have reported successful NIST SP 800-22 results from deterministic seeds. GAN- and WGAN-GP-based designs have aimed to generate statistically strong bitstreams under different training strategies [22,24,26,27,28]. Reinforcement learning (RL)-based approaches have also treated randomness generation as a sequential decision-making problem and evaluated LSTM- or agent-based generation structures [25,29,30]. These studies are valuable because they demonstrate the potential use of learning-based models in PRNG design. However, from the perspective of DNA-based processes, most of these approaches operate directly in the bit domain. They do not treat DNA-specific measures, such as GC balance, homopolymer limit, short-range dependencies, and local motif behavior, as embedded components of the generation process [3,4,5,6]. In addition, the dependence of many methods on training data, long optimization procedures, or high computational cost may introduce further limitations in terms of data dependence and reproducibility [22,23,24,25,26,27,28,29,30,31,32,33].

Hybrid systems involving DNA coding constitute a broad literature, particularly in image encryption, key generation, and multilayer security mechanisms [31,34,35,36,37,38]. In these studies, DNA bases are generally not used as the primary output space in which randomness is directly generated. Instead, they are used as an intermediate layer in which binary data are symbolically represented or transformed during encryption. Hybrid structures that combine dynamic DNA coding schemes, multi-rule DNA mappings, chaotic maps, and deep learning components have made important contributions in terms of encryption diversity, key variability, and implementation flexibility [12,13,31,34,35,36,37,38]. However, the main objective of these studies is generally not to develop an independent, DNA-local PRNG core that manages constraints during generation. Therefore, although the DNA coding and encryption-oriented literature is functional within its own application domain, it does not fully address the need for an independent and constraint-aware randomness generation core that can be directly integrated into DNA-based processes.

Transformer-based PRNG studies represent a more recent research direction in this field. Recent studies have shown that decoder-only Transformer architectures can learn the output behavior of classical PRNG families and model certain algorithmic regularities [8,11]. For example, Transformer-based approaches have been used to model the output patterns of classical generators such as the linear congruential generator (LCG) and Mersenne Twister, to evaluate bit-level randomness test performance, and to represent certain structural regularities [8]. Similarly, findings related to modular generalization and residue number system (RNS)-based interpretability indicate that Transformer architectures can capture specific algorithmic patterns [11]. Nevertheless, existing Transformer-based PRNG studies remain largely limited to the bit domain. Direct generation in the A/C/G/T alphabet, GC balance and homopolymer control during generation, regulation of short-range DNA dependencies, and dynamic DNA-to-bit rule selection are not the main focus of these studies. Moreover, this research line does not compare regimes with and without training data under the same DNA-local PRNG core.

Taken together, the existing literature provides important contributions to PRNG design, DNA coding, hybrid encryption, and Transformer-based modeling. However, for DNA-based workflows, limited attention has been given to a PRNG framework that directly generates in the DNA alphabet, manages DNA-specific constraints during generation, derives the bitstream through dynamic DNA-to-bit rule selection, and compares data-supported and data-independent conditions under the same core. Studies that combine general-purpose randomness tests and DNA-specific structural measures within the same validation protocol are also limited. This study addresses this gap through a dual-head Transformer-based, DNA-local, and constraint-aware PRNG core. The main differences between representative studies and the proposed method are summarized in Table 1. A broader comparison and additional reporting dimensions are provided in Appendix A, Table A1.

Table 1 shows that the difference of the proposed method does not arise from a single component. Rather, it results from the joint treatment of DNA-local generation, constraint management during generation, dynamic DNA-to-bit rule selection, a reference-free generation setting, and an integrated validation protocol.

## 3. Materials and Methods

### 3.1. Overview of the Proposed Method

**Figure 1:**
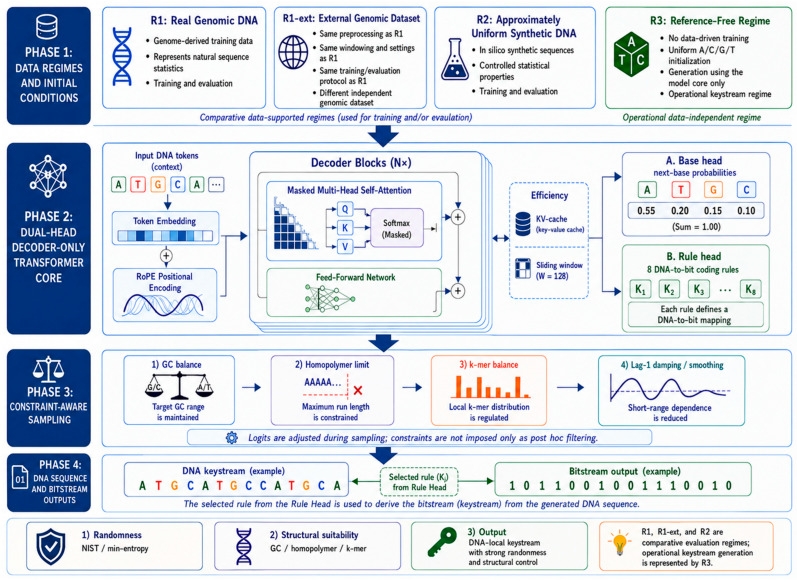
Overall workflow of the proposed DNA-local PRNG framework. The figure summarizes the data-supported and reference-free regimes, the RoPE-based dual-head decoder-only. Transformer core, constraint-aware DNA sampling, and the joint generation of DNA keystream and bitstream outputs. Arrows indicate the processing flow between modules.

The proposed framework is designed as a constraint-aware and reproducible PRNG core that generates outputs directly in the A/C/G/T alphabet for DNA-based workflows. Unlike conventional bit-first approaches, generation is performed in the DNA domain, and the corresponding bitstream is derived within the same processing pipeline. For this purpose, the proposed computational core is based on a dual-head decoder-only Transformer architecture. The first output head generates the probability distribution for the next DNA base, whereas the second output head models the selection behavior among eight equivalent DNA-to-bit coding rules. Thus, DNA sequence generation, rule selection, and bitstream derivation are treated as interacting components of the same generation process. The general framework shown in Figure 1 evaluates the same architectural core under three complementary generation regimes.

The R1 regime represents the data-supported setting trained on real genomic DNA. The R2 regime represents the controlled setting trained on approximately uniform synthetic DNA. The R3 regime represents the reference-free setting that operates without any training data. This structure makes it possible to examine the effect of different data conditions on generation behavior. In particular, the R3 regime provides a control setting for evaluating the model behavior independently of learned distributions. An additional external validation setting is also included in the same pipeline to assess dependence on a single real genomic source more explicitly.

The general workflow consists of four main phases. First, the data regime and initial conditions are defined. Second, the RoPE-based dual-head decoder-only Transformer core produces base-head and rule-head outputs. Third, constraint-aware sampling regulates GC balance, homopolymer limits, local k-mer behavior, and short-range dependency during generation. Finally, the DNA keystream, selected rule trace, corresponding bitstream, and evaluation outputs are obtained together. At the end of this process, the DNA sequence, the selected rule trace, and the corresponding bitstream are produced together. Therefore, the proposed method is not only a generator of bit-level randomness. It is also an integrated generation core that manages DNA-level structural compatibility in a traceable manner.

To ensure reproducibility, all regimes use a seed-controlled and traceable execution protocol. Data selection, initial context construction, base sampling, and rule selection are governed by the same randomness-control logic. In the default configuration, no external cryptographically secure pseudorandom number generator (CSPRNG), extractor, or key-derivation function is used. This choice ensures that the reported bit-level and DNA-level statistics directly reflect the behavior of the proposed Transformer core and the constraint-aware sampling mechanism. The generated DNA sequences, bitstreams, rule traces, and run metadata are stored as separate outputs to support the reproducibility and traceability of the experiments.

### 3.2. Data Regimes and Generation Modes

The proposed DNA-local PRNG framework was evaluated under three generation regimes to examine how the same architectural core behaves under different data conditions. The R1 regime represents the data-supported setting trained on real genomic DNA. The R2 regime represents the controlled setting trained on approximately uniform synthetic DNA. The R3 regime represents the reference-free setting that operates without any external training data. This design aims to separate the observed output behavior from the effects of training data, model architecture, and generation-time constraint-aware sampling. In all regimes, the same decoder-only Transformer core and the same output generation pipeline are preserved. The main differences between regimes are the data source and the relationship of the model to training.

In the R1 regime, the complete genome of Escherichia coli strain K-12 substrain MG1655 was used as the real genomic source [14]. FASTA headers were removed, non-A/C/G/T symbols were cleaned, and the first 1,000,000 bases of the cleaned sequence were used as the source pool. In each independent run, the source pool was divided into non-overlapping 1000-base blocks. From these blocks, 25 blocks were selected using a run_seed-controlled random sampling procedure, resulting in a 25,000-base sub-corpus for each run. The selected blocks were used only within the corresponding run and did not overlap with the sub-corpora of other runs. The resulting sub-corpus was divided into 20,000 bases for training and 5000 bases for validation. In addition, an independent genomic validation setting, R1-ext, was generated using the complete genome of Bacillus subtilis subsp. subtilis strain 168 [39]. This setting was not treated as a new main regime, but as an external validation extension of R1 on an independent genomic source. The same cleaning procedure, source-pool length, non-overlapping block sampling scheme, training/validation split, and production pipeline were preserved. This additional setting was used to assess more clearly whether the method depends on a single real genomic source.

In the R2 regime, the same experimental structure as R1 was preserved. However, instead of a real genomic source, approximately uniform synthetic DNA was used [15]. The synthetic source pool consisted of a 1,000,000-base DNA sequence generated in Python by uniform sampling over the A/C/G/T alphabet. As in R1, each run selected 25 non-overlapping 1000-base blocks, forming a 25,000-base sub-corpus. This sub-corpus was then split into 20,000 bases for training and 5000 bases for validation. The use of the same data size and the same experimental settings in R1 and R2 enabled comparison between effects arising from real genomic patterns and the generation behavior observed under an approximately uniform distribution.

The R3 regime was defined as a reference-free generation setting designed to remove the effect of training data. In this regime, the model parameters were not optimized on any genomic or synthetic training corpus. Generation was initialized with a uniform initial context over the A/C/G/T alphabet and continued using the same autoregressive production pipeline. Therefore, R3 is not a third trained model variant. Instead, it is a data-independent comparison setting used to evaluate generation behavior after removing the influence of learned distributions.

In all regimes, output generation was carried out through the same basic pipeline. The generation process started from an initial sequence with a fixed context length. At each step, the model produced a probability distribution for the next DNA base and a separate distribution for DNA-to-bit rule selection. The selected DNA base was appended to the sequence, and the corresponding two-bit output was derived using the selected rule. In each run, a 500,000-base DNA sequence and an approximately 1 Mbit bitstream were obtained. The DNA sequence, bitstream, rule trace, and run metadata were stored as separate files. This structure supports reproducibility and makes each output component traceable. In the training regimes, the open non-overlapping block-based sampling design was used to prevent overlap within the sub-corpus. The independent genomic validation setting provides an additional control under the same pipeline to assess dependence on a single data source. The general workflow applied under R1–R3 is summarized in Algorithm 1.
**Algorithm 1.** Proposed DNA-local PRNG generation workflow under R1, R1-ext, R2, and R3**Input:** Generation regime ∈ {R1, R1-ext, R2, R3}, context length W, target generation length L, run_seed_, model configuration, and sampling parameters. **Output:** Generated DNA sequence X, rule trace R, and corresponding bitstream Y.Select the generation regime: R1, R1-ext, R2, or R3.**If** the regime is R1, R1-ext, or R2:
Prepare the relevant source pool: real genomic DNA for R1, independent genomic DNA for R1-ext, and approximately uniform synthetic DNA for R2.Select non-overlapping 1000-base blocks from the source pool under run_seed control.Construct a 25,000-base sub-corpus.Split the sub-corpus into training and validation partitions.Calibrate the MiniGPTDualHead model on the relevant sub-corpus.**If** the regime is R3:
Do not use training data.Construct a uniform initial context.Set the initial context as X; initialize the rule trace R and bitstream Y as empty sequences.**For** t = 1, …, L:
Compute the base-head and rule-head outputs.Select the next DNA base using constraint-aware sampling.Select the DNA-to-bit rule.Append the selected base to X.Append the selected rule to R.Derive the two-bit output from the selected base and rule, and append it to Y.
**Return** the generated DNA sequence, rule trace, and bitstream, excluding the initial context.

### 3.3. Dual-Head Decoder-Only Transformer Architecture

**Figure 2:**
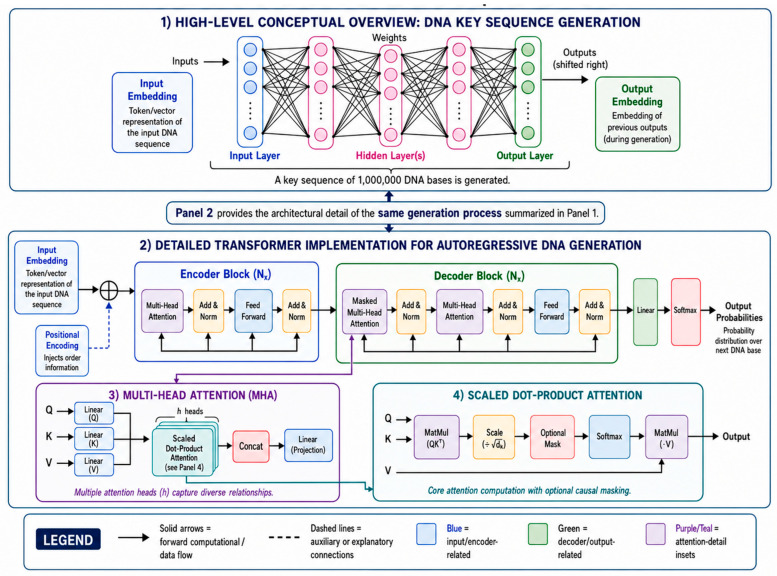
Dual-head decoder-only Transformer core.

The proposed DNA-local PRNG core is based on a lightweight decoder-only Transformer architecture designed for autoregressive sequence generation [7]. This choice follows from the nature of the problem. The task is not a source-to-target transformation, but a one-directional DNA sequence generation problem conditioned on past context. Therefore, the encoder block and cross-attention layers used in classical encoder–decoder Transformer architectures are not employed in this study. The proposed core uses causal self-attention over the previous context and produces probability distributions for both the next DNA base and the DNA-to-bit rule selection. The general structure of the proposed dual-head decoder-only Transformer core is shown in Figure 2.

The model processes a sliding DNA context, applies Rotary Positional Embedding (RoPE)-based causal self-attention, and produces two output distributions: the base-head distribution for the next DNA base and the rule-head distribution for DNA-to-bit rule selection. DNA-specific structural constraints are not embedded directly into the learned weights of the Transformer core. Instead, they are managed through the constraint-aware sampling layer applied during generation. This design provides a simpler core focused on DNA sequence generation while also limiting computational cost. The proposed model consists of three Transformer blocks, four attention heads, a 128-dimensional embedding space, and a 128-base sliding context window. The expansion ratio in the feed-forward layer is set to 4d_model. To reduce latency and memory cost during long-sequence generation, a key–value (KV) cache is used. The architecture used in this study is not a direct copy of the original Transformer model [7]. It is a lightweight core restructured for DNA-local generation and transition to the bit domain. The main architectural differences between the original Transformer and the proposed DNA-PRNG core are summarized in Table 2. A more detailed comparison is provided in Appendix A, Table A2.

The input vocabulary is restricted to the DNA alphabet. This alphabet consists of four bases$:\;\Sigma =\left\{A,C,G,T\right\},|\Sigma |=4$. Each base $b_{t}\in \Sigma$ is represented as a separate token identity and mapped to a learnable embedding space. Let W denote the context-window length. At time step t, the input sequence is defined as: $s_{t}=\left(b_{t-W+1},\ldots ,b_{t}\right).$ The initial representation matrix is given in Equation (1):

(1) $$H^{\left(0\right)}=\left[Embed\left(b_{t-W+1}\right),\ldots ,Embed\left(b_{t}\right)\right]\in R^{W\times d_{model}}.$$

Positional information is not used as a simple vector directly added to the embedding vectors. Instead, relative positional structure is represented by applying RoPE to the query and key vectors in the attention layer. This choice is appropriate for autoregressive generation and sliding-window operation, because it allows relative positional relationships to be modeled more naturally. Each Transformer block consists of a residual multi-head self-attention (MHSA) module with pre-layer normalization (pre-LayerNorm) and a feed-forward network (FFN) with Gaussian Error Linear Unit (GELU) activation. For block l, the transformations are defined in Equations (2)–(4):

(2) $$X^{\left(l\right)}=LN(H^{\left(l-1\right)}),$$

(3) $${\tilde{H}}^{\left(ı\right)}=H^{\left(l-1\right)}+{MHSA}^{\left(l\right)}(X^{\left(l\right)})$$

(4) $$H^{\left(l\right)}{=\tilde{H}}^{\left(ı\right)}+{FFN}^{\left(l\right)}\left(LN\left({\tilde{H}}^{\left(ı\right)}\right)\right),\;\;l=1,\ldots ,L,$$

In the multi-head self-attention layer, the query, key, and value projections are computed as: $Q=XW_{Q},\;K=XW_{K},\;V=XW_{V}.$ RoPE is then applied to the query and key vectors as shown in Equation (5):

(5) $$\tilde{Q}=RoPE\left(Q\right),\;\;\;\;\tilde{K}=RoPE\left(K\right).$$

The scaled dot-product attention under a causal mask is then computed as shown in Equation (6):

(6) $$Attention\left(Q,K,V\right)=softmax\left(\frac{{\tilde{Q}\tilde{K}}^{⊤}}{\sqrt{d_{h}}}+M_{causal}\right)V.$$

Here $d_{h}=d_{model}/h=32$ and $M_{causal}\;$denotes the causal attention mask that suppresses access to future positions. The feed-forward subnetwork is defined by Equation (7):

(7) $$FFN\left(z\right)=W_{2}GELU\left(W_{1}z+b_{1}\right)+b_{2}$$

The distinguishing architectural feature of the proposed core is its dual-head structure. The final hidden representation is directed to two separate output heads. Let the final representation vector be: $z_{t}\in R^{d_{model}}$. The logits produced by the base head and rule head are given in Equation (8):

(8) $$o_{t}^{base}=z_{t}W_{base}\in R^{4},\;\;o_{t}^{rule}=z_{t}W_{rule}\in R^{8}.$$

The corresponding probability distributions are obtained through the softmax transformation in Equation (9):

(9) $$p_{t}\left(b\right)=softmax{\left(o_{t}^{base}\right)}_{b}\;,\;\;q_{t}\left(r\right)=softmax{\left(o_{t}^{rule}\right)}_{r}.$$

Here, the base head produces the probability distribution for the next DNA base. The rule head models the selection behavior over the eight equivalent DNA-to-bit coding rules used to derive the bitstream from the generated DNA sequence. Thus, the proposed architecture operates not only as a DNA sequence generator, but also as a joint generation structure that links the DNA and bit domains. This design also helps limit secondary regularities that may arise from using a single fixed conversion rule. An important architectural point is that DNA-specific structural constraints are not directly embedded into the learned network weights. Instead, GC balance, homopolymer control, short-range dependency suppression, and local n-gram regulation are applied through the constraint-aware sampling layer operating on the probabilities produced by the Transformer core. In other words, the Transformer core is responsible for probability generation, while the sampling layer transforms these probabilities into DNA sequences that are structurally more compatible with DNA-specific requirements. DNA-specific controls are applied at generation time, and the corresponding sampling terms are described in detail in Section 3.5.

### 3.4. Learning Objective and Regime-Specific Training Strategy

In the proposed DNA-local PRNG framework, the learning step is applied only in data-supported generation settings. The R1 and R1-ext regimes are calibrated on real genomic DNA, whereas the R2 regime is calibrated on approximately uniform synthetic DNA. In contrast, the model is not optimized on any training corpus in the R3 regime. R3 is therefore treated as a data-independent reference setting in which the effect of learned distributional information is removed. For this reason, the learning objective defined in this section applies only to R1, R1-ext, and R2. No training loss or convergence curve is defined for R3.

In the R1, R1-ext, and R2 regimes, the learning objective has two main goals. The first is to calibrate the base-head output to model the next DNA base. The second is to guide the rule-head output toward balanced selection behavior over the eight equivalent DNA-to-bit coding rules, without collapsing to a single rule. Following the operation order in the code-level implementation, the base-head cross-entropy loss is first computed. Then, the soft target distribution for the rule head is constructed using the base probabilities produced by the base head and the DNA-to-bit rule matrix. Finally, the rule-head soft-target cross-entropy loss and the uniformity Kullback–Leibler (KL) regularization term are computed. The total training objective is defined as the weighted sum of these three components. Let the four-class probability distribution produced by the base head at time step t be denoted by $p_{t}(\cdot )$. The base-head loss is given in Equation (10):

(10) $$L_{base}=\;-\frac{1}{T}\sum_{t=1}^{T}\log p_{t}(b_{t+1}\;|b_{\leq t})$$

The soft-target cross-entropy loss for the rule head is defined in Equation (11):

(11) $$L_{rule}=\;-\frac{1}{T}\sum_{t=1}^{T}\sum_{r\in R}\tilde{y_{t}}\left(r\right)\;\log q_{t}\left(r\right)$$

To prevent the rule-head distribution from collapsing to a small subset of the eight rules, a uniformity regularization term is used. This term is given in Equation (12):

(12) $$L_{unif}=\;\frac{1}{T}\sum_{t=1}^{T}D_{KL}(q_{t}∥U_{8})$$

The total training objective is given in Equation (13):

(13) $$L=L_{base}+\;\lambda_{rule}L_{rule}+\;\beta_{unif}L_{unif}$$

Here, T denotes the total number of autoregressive time steps included in the loss computation. The set R={0,1,…,7} denotes the eight DNA-to-bit coding rules. The term $p_{t}\left(.\right)\;$is the next-base probability distribution produced by the base head. The term $q_{t}$ is the probability distribution over the eight rules produced by the rule head. The soft target distribution $\tilde{y_{t}}$ is constructed using the base-head probabilities and the DNA-to-bit rule matrix. The term $U_{8}$ denotes the uniform distribution over the rule set. The coefficients $\lambda_{rule}$ and $\beta_{unif}$ control the relative contributions of the soft-target rule loss and the uniformity regularization term to the total loss.

The soft target distribution $\tilde{y_{t}}$ is constructed by considering the expected 0/1 balance of the two-bit outputs under the eight DNA-to-bit rules. This structure does not force the rule head to predict a single fixed rule. Instead, it assigns higher weights to rules that are expected to support more balanced bit-level output. The additional uniformity regularization term limits the collapse of the rule-head distribution to a narrow subset of rules. Together, the soft-target rule loss and the uniformity regularization aim to increase diversity in rule usage during bitstream derivation and to reduce secondary regularities that may arise from using a single fixed DNA-to-bit conversion rule.

Training in the R1, R1-ext, and R2 regimes was performed for a single epoch using the AdamW optimization algorithm. The batch size was set to 256, the learning rate to 3 × 10^−4^, and the weight decay to 0.01. The rule-head regularization coefficients were set as $\lambda_{rule}=0.2$ and $\beta_{unif}=0.2$ and the soft-target temperature was set as $\tau_{rule}=0.25.$ In each trained regime, the model was calibrated on the training sub-corpus, and the validation bits-per-base value was monitored on a separate validation sub-corpus. This separation allows direct fit to the training data and validation behavior to be evaluated separately.

In this study, the training stage is used as a limited calibration step for data-supported regimes, rather than as a long-term convergence optimization process. Therefore, for R1, R1-ext, and R2, convergence is not interpreted through multi-epoch training curves. Instead, it is evaluated together with the validation bits-per-base value monitored on the separate validation sub-corpus, variability across independent runs, and the R1-ext external validation results. The risk of overfitting and memorization is also assessed using the single-epoch training design, the separate validation sub-corpus, and the exact-match k-mer leakage control against the reference or training corpora. In the R3 regime, no training or optimization step is performed. Therefore, training convergence and overfitting are not defined for this regime.

Although the network weights are optimized on data in the R1, R1-ext, and R2 regimes, DNA-specific structural controls such as GC balance, homopolymer limit, short-range dependency suppression, and local n-gram regulation are not embedded directly into the loss function. These constraints are managed through the constraint-aware sampling mechanism applied at generation time. This separation makes it possible to evaluate more clearly the learning capacity of the architectural core and the effect of the constraint-aware generation strategy.

The R3 regime is a data-independent generation setting defined to exclude the effect of training data. In this regime, the model parameters are not optimized on any genomic or synthetic training corpus. Generation starts from a uniform initial context and proceeds through the same autoregressive generation pipeline. Therefore, R3 is not treated as a third trained model variant. Instead, it is used as a comparative reference setting that removes the influence of learned distributions. Since no optimization step is applied, training loss, validation bits-per-base, and training convergence are not reported for R3. This setting is used to evaluate how the architectural core and the constraint-aware sampling layer behave without training data.

Reproducibility across all regimes is ensured through a single seed-controlled randomness logic. In trained regimes, this logic controls source sub-corpus selection, initial context construction, and generation steps. In R3, it controls the uniform initial context and the entire generation process. In the default configuration, no external CSPRNG, extractor, or key-derivation function is used. This choice ensures that the reported results originate from the joint behavior of the proposed dual-head decoder-only Transformer core and the constraint-aware sampling layer, rather than from an external post-processing module.

### 3.5. Constraint-Aware DNA Sampling

In the proposed framework, the Transformer core produces a probability distribution for the next DNA base at each autoregressive step. A constraint-aware sampling layer before direct sampling from this distribution manages DNA-specific structural compatibility. Thus, GC balance, homopolymer control, short-range dependency suppression, and local n-gram regulation are not treated as post hoc corrections applied after sequence generation. Instead, they are handled as in-process mechanisms that reshape the sampling distribution at each generation step. This design functionally separates the probability-generation capacity of the learned Transformer core from the sampling layer that enforces DNA-compatible output generation. As defined in Section 3.3, at time step t, the base head produces a raw logit vector over the four DNA bases. Let this vector be denoted by: $o_{t}^{base}\in R^{4}$. Instead of sampling directly from these logits, an initial temperature-scaled base distribution is first obtained as: $p_{t}^{0}\left(b\right)=softmax\left(\frac{o_{t}^{base}\left(b\right)}{\tau }\right),\;b\in \Sigma$. The constraint-adjusted logit vector is then defined in Equation (14):

(14) $$\tilde{z_{t}}\left(b\right)=\log p_{t}^{0}\left(b\right)+\delta_{t}^{GC}\left(b\right)+\delta_{t}^{lag}\left(b\right)+\delta_{t}^{ng}\left(b\right)+m_{t}^{HP}\left(b\right),\;\;b\in \Sigma$$

Here, $p_{t}^{0}\left(b\right)$ denotes the initial base probability obtained from the base-head logits after temperature scaling. The parameter τ is the temperature used to construct this initial distribution. The term $\delta_{t}^{GC}\left(b\right)$ denotes the soft correction for GC balance. The term $\delta_{t}^{lag}\left(b\right)$ denotes the short-range dependency suppression term. The term $\delta_{t}^{ng}\left(b\right)$ denotes the local n-gram regulation term. The term $m_{t}^{HP}\left(b\right)$ denotes the mask used for enforcing the homopolymer limit. In this study, n-gram regulation is implemented through low-order bigram and 3-mer smoothing components. It aims to limit excessive concentration of local transitions or short motifs. GC balancing is applied as a soft steering term according to the target base distribution corresponding to the desired GC ratio. Let the target GC ratio be: $\rho^{*}=0.5$. The target base distribution is defined as: $\pi_{A}=\pi_{T}=\frac{1-\rho^{*}}{2},\;\pi_{C}=\pi_{G}=\frac{\rho^{*}}{2}$. During generation, the remaining quota or deviation from the target distribution is considered. The GC-guided logit correction for candidate base b is given in Equation (15):

(15) $$\delta_{t}^{GC}\left(b\right)=\alpha_{t}\log\left(\frac{max(\varepsilon ,\;\;{\hat{\pi }}_{t}(b))}{max(\varepsilon ,\;\;\pi_{b})}\right)$$

Here, ${\hat{\pi }}_{t}(b)$ denotes the remaining target proportion or soft balancing ratio for base b at the relevant generation step. The term $\pi_{b}$ denotes the target base proportion, and ε is a small positive constant used for numerical stability. The coefficient $\alpha_{t}$ controls the strength of the GC-balancing effect as generation progresses. This term does not apply GC control as a hard post hoc correction. Instead, it acts as a guiding preference embedded into the sampling distribution.

Homopolymer control is not treated as a soft preference. It is handled as a hard feasibility condition. Let $r_{t}$ denote the current consecutive repeat length of the same base at the end of the prefix, and let $H_{max}$ denote the maximum allowed homopolymer length. The homopolymer mask is defined in Equation (16):

(16) $$m_{t}^{HP}\left(b\right)=\left\{\begin{array}{cc} -\infty , & if\;b=b_{t}\;and\;r_{t}\geq H_{max}\;\\ 0, & otherwise. \end{array}\right.$$

In this study, $H_{max}=5$. Thus, candidate bases that would exceed the allowed homopolymer limit are directly removed from the sampling support. This mechanism prevents the formation of long single-base repeats during generation and limits homopolymer structures that may increase error risk in synthesis or sequencing workflows. Short-range dependency suppression is applied to reduce the tendency of the same base to become locally dominant in consecutive or nearby transitions. The general lag-1 correction is given in Equation (17):

(17) $$\delta_{t}^{lag}\left(b\right)=\alpha_{lag}\log\left(1-\eta_{lag}1\left[b=b_{t}\right]\right)$$

Here, $\eta_{lag}$ is the suppression coefficient for repeated selection of the same base, and $\alpha_{lag}$ denotes the relative strength of this correction. This term helps prevent the same base from becoming excessively dominant in one-step local transitions. Local n-gram regulation is applied according to the observed counts of low-order motifs that the candidate base would form with the current prefix. The general n-gram correction term is given in Equation (18):

(18) $$\delta_{t}^{ng}\left(b\right)={-\alpha }_{ng}\log\left(\frac{c_{t}\left(g_{b}\right)+1}{E_{t}+1}\right)$$

Here, $g_{b}$ denotes the bigram or 3-mer motif that candidate base b would form with the current prefix. The term $c_{t}\left(g_{b}\right)$ is the number of times this motif has been observed so far in the generated prefix. The term $E_{t}$ denotes the expected average count for the corresponding n-gram space. The coefficient $\alpha_{ng}$ controls the strength of the correction. This term softly suppresses excessive concentration of local motifs and contributes to a more balanced short-motif distribution. After all correction terms are combined, the final sampling distribution is obtained by normalized exponentiation, as shown in Equation (19):

(19) $$\tilde{p_{t}}\left(b\right)=\frac{exp(\tilde{z_{t}}\left(b\right))}{\sum_{b^{\prime }\in \Sigma }exp(\tilde{z_{t}}\left(b^{\prime }\right))}$$

The next DNA base is then sampled from this distribution: $b_{t+1}\sim \;Categorical(\tilde{p_{t}})$. When necessary, the support set can also be restricted by top-k pruning. However, in the default configuration, the main decision mechanism is based on the probability distribution reshaped directly by constraint-adjusted logits. In this respect, the proposed sampling strategy differs from post-generation repair approaches that modify the completed sequence afterward. DNA-specific compatibility is managed at the probability-distribution level during every generation step. The general constraint-aware sampling workflow is summarized in Algorithm 2.
**Algorithm 2.** Constraint-aware sampling**Input:** Base-head logits $o_{t}^{base}$, current prefix, temperature τ, GC target, homopolymer limit, lag-1 parameters, and n-gram parameters.
**Output:** Selected next DNA base $b_{t+1}$.**1.** Obtain the temperature-scaled initial base distribution $p_{t}^{0}$ from the base-head logits.**2.** Compute the GC correction term according to deviation from the GC target.**3** Compute the lag-1 correction term according to short-range transition dominance.**4.** Compute the n-gram correction term according to local n-gram concentration.**5.** Mask candidate bases that would exceed the homopolymer limit.**6.** Combine all correction terms to construct the constraint-adjusted logit vector.**7.** If necessary, restrict the support set using top-k pruning.**8.** Normalize the constraint-adjusted logits with temperature to obtain the final sampling distribution.**9.** Sample the next DNA base from the final distribution.**10.** Append the selected base to the DNA output sequence.**11.** Store the updated state for the next generation step.

The constraint-aware sampling layer is applied according to the same basic principle in R1, R1-ext, R2, and R3. Therefore, the observed differences among regimes do not originate from different constraint definitions. Instead, they arise from how the base-head probabilities are formed under real-data, synthetic-data, or data-independent initial conditions. In other words, this layer acts as a common regulatory wrapper across all regimes and helps separate the effect of the data source from the effect of the sampling mechanism. While the Transformer core produces probabilities, the constraint-aware sampling layer transforms these probabilities into DNA-compatible generation behavior. Dynamic DNA-to-bit rule selection and bitstream derivation are defined separately in the following section and are treated as monitored output components in the evaluation protocol.

### 3.6. DNA-to-Bit Mapping and Construction of Output Streams

The proposed framework is not designed only as a structure that generates DNA sequences. A second objective is to combine DNA-local generation with a randomness output that can be used in the bit domain within the same processing pipeline. Therefore, the DNA base obtained from the constraint-aware sampling layer defined in Section 3.5 is converted into a binary output at each generation step through dynamic DNA-to-bit rule selection. In this way, the output is obtained not only as a DNA sequence, but also as a traceable bitstream that does not depend on a single fixed mapping rule. As defined in Section 3.3, the model operates with eight equivalent DNA-to-bit coding rules. This rule set is denoted by R and is given in Table 3. Each rule defines a different mapping between the four bases in the DNA alphabet and two-bit outputs. Accordingly, for each r∈R, the conversion function is defined as: $\phi_{r}:\Sigma ⟶{\left\{0,1\right\}}^{2}$. Since the same DNA base may correspond to different two-bit outputs under different rules, the behavior of the resulting bitstream depends not only on the generated DNA sequence, but also on the selected rule sequence.

During generation, rule selection is performed adaptively using the constraint-adjusted base distribution obtained in Section 3.5 and the DNA-to-bit rule matrix. For each rule, the expected two-bit output balance is computed, and rules that are closer to the 0/1 balance are assigned higher selection probabilities. At time step t, the adaptive rule-selection distribution used during generation is defined in Equation (20):

(20) $$\begin{array}{c} E_{t}\left(r,j\right)=\sum_{b\in \Sigma }{\tilde{p}}_{t}\left(b\right)M_{r}\left(b,j\right),\;\;\;\;\;\;j\in \left\{1,2\right\}\\ s_{t}^{rule}\left(r\right)=1-\frac{\left|E_{t}\left(r,1\right)-0.5\right|+\left|E_{t}\left(r,2\right)-0.5\right|}{2}\\ \pi_{t}^{rule}\left(r\right)=softmax\left(\frac{s_{t}^{rule}\left(r\right)}{\tau_{rule}}\right),\;\;\;\;\;\;r\in R \end{array}$$

Here, $M_{r}\left(b,j\right)$ denotes the value of base b at bit position j under rule r. The term $E_{t}\left(r,j\right)$ denotes the expected bit value produced by the corresponding rule under the current base distribution. The score $s_{t}^{rule}\left(r\right)$ indicates how close the rule is to a balanced two-bit output, and $\pi_{t}^{rule}\left(r\right)$ denotes the adaptive rule-selection distribution used during generation. In implementation, this basic balance score can be supported, when necessary, by a mild diversity term that reduces concentration of recently used rules. However, the main selection principle is to preserve the expected two-bit 0/1 balance under the current base distribution. The selected rule rtr_trt is applied to the DNA base sampled at the same generation step. The corresponding two-bit output is obtained as shown in Equation (21):

(21) $$y_{t}=\phi_{rt}\left(b_{t+1}\right)\in {\left\{0,1\right\}}^{2}$$

Accordingly, the final DNA output and the corresponding bitstream are defined as: $X=\{b_{1},b_{2},\ldots ,b_{N}\}$ and $Y=y_{1}∥y_{2}∥\cdots ∥y_{N}$. Here, the operator ∥ denotes concatenation of consecutive two-bit blocks. Since each base is converted into a two-bit output, a DNA sequence of length N produces a bitstream of length 2N. For example, a 500,000-base DNA output corresponds to an approximately 1,000,000-bit stream.

The main purpose of dynamic rule selection is to prevent the bit-domain behavior from becoming overly dependent on a single fixed DNA-to-bit mapping rule. If one conversion rule is fixed throughout the entire generation process, local concentration of some base patterns may induce secondary regularities in the bitstream. In contrast, adaptive selection among eight equivalent rules supports a more balanced and diverse bit-level output behavior. Therefore, rule selection is not treated as a simple auxiliary labeling step, but as an active component that regulates diversity during the transition from the DNA domain to the bit domain.

In the R1, R1-ext, and R2 regimes, the rule head is calibrated using the soft-target loss and uniformity regularization described in Section 3.4. However, in the default generation pipeline, rule selection is performed through the adaptive selection mechanism computed from the final base distribution and the rule matrix. In the R3 regime, because model parameters are not trained, rule selection is carried out without the influence of learned distributional information. It is instead performed under data-independent initial conditions and the same adaptive generation principle. Thus, the bitstream obtained in R3 reflects the joint behavior of the architectural core, the constraint-aware sampling layer, and the adaptive DNA-to-bit mapping mechanism, rather than a rule behavior learned from training data.

In the proposed framework, the DNA sequence, the corresponding bitstream, and the selected rule trace are stored as separate outputs for each generation run. This structure enables the DNA output, bitstream behavior, and rule-usage pattern to be examined together within the same run. It also supports comparison of rule usage across regimes, analysis of distributional effects in the bit domain, and experimental reproducibility. In the default configuration, the bitstream is derived directly from the proposed DNA-local generation pipeline. No external extractor, key-derivation function, or additional post-processing layer is used. This choice ensures that the reported bit-level statistics originate from the joint production behavior of the dual-head decoder-only Transformer core, the constraint-aware sampling layer, and the adaptive DNA-to-bit rule-selection mechanism.

## 4. Results

### 4.1. Evaluation Setup and Reproducibility Summary

The proposed DNA-local Transformer-based PRNG framework was evaluated under the R1, R2, and R3 generation regimes to compare the behavior of the same architectural core under different data and training conditions. In addition, to examine whether the method depends on a single real genomic source, the R1 protocol was applied to an independent genomic data source as the R1-ext external validation setting. R1-ext is not a new main generation regime, but an external validation extension of R1 on independent genomic data.

For each setting, 10 independent generation runs were performed. In each run, a 500,000-base DNA sequence and the corresponding 1,000,000-bit stream were generated. The same architectural core, autoregressive generation pipeline, constraint-aware sampling mechanism, and adaptive DNA-to-bit rule-selection strategy were preserved across all settings. In R1, R1-ext, and R2, the model was calibrated under the corresponding data condition. In R3, no training or optimization step was applied.

To support reproducibility, sub-corpus selection, initial context construction, base sampling, and rule selection were controlled through a single PyTorch-based random generator. Generator initialized with the run-specific run_seed. The seed values, generated DNA sequences, bitstreams, rule traces, and run metadata were recorded. Computational measurements, including wall-clock time, CPU time, and memory usage, were collected under CPU-only conditions to ensure comparability across regimes. The experiments were conducted on Windows 11 Pro 64-bit, an Intel Core i7-10750H processor, 16 GB random-access memory (RAM), and Python 3.12.3. The main software packages used in the implementation were NumPy version 1.24.4, SciPy version 1.12.0, PyTorch version 2.0.0+cpu, and Biopython version 1.83. GPT-5.5 was used for language editing, limited text refinement, and visual/figure refinement. The run parameters and output-file access information are provided in the detailed reproducibility description and the Data Availability Statement. All generation scripts, run parameters, run_seed values, generated DNA sequences, bitstreams, rule traces, metadata files, and run-level evaluation outputs were also shared in the GitHub repository. This structure supports independent inspection of the reported results and regime-specific outputs.

The results were not reported from a single representative run. Instead, they were summarized across 10 independent runs. Where applicable, the result tables include the mean ± sample standard deviation (SD), 95% confidence interval (CI), observed minimum–maximum range, and the number of successful runs. For individual sequence-test results within NIST SP 800-22, the threshold was set to *p* ≥ 0.01. Results across multiple runs were evaluated together with pass counts and distributional summaries. Bit-level statistical randomness, DNA-specific structural compatibility, rule-usage behavior, multi-stream independence, robustness, component contributions, computational cost, and theoretical complexity are presented in the following subsections.

### 4.2. Bit-Level Randomness Evaluation

#### 4.2.1. NIST SP 800-22 Tests

The bitstreams derived from the proposed DNA-local PRNG framework were evaluated using the NIST SP 800-22 test suite at the significance level of α = 0.01 [16,17]. For each of the R1, R1-ext, R2, and R3 settings, 10 independent bitstreams were analyzed, and each stream was generated with a length of 1,000,000 bits. The tests were applied to the binary outputs obtained from the generated DNA sequences through adaptive DNA-to-bit rule selection.

Table 4 presents, for the 15 main tests, the mean ± sample SD, Student-t-based 95% CI, observed minimum–maximum range, and pass count of the *p*-values obtained from 10 independent streams. The mean *p*-values and CIs are reported as descriptive statistics. The pass decision was determined separately for each stream using the criterion *p* ≥ 0.01. Therefore, no superiority ranking among settings was made based on the magnitude of mean *p*-values.

For tests that produce multiple *p*-values, the same reporting rule was applied across all settings to construct a compact summary table. Table 4 reports the M = 1000 result for the Block Frequency test, the selected 000000001 template for the Non-overlapping Template test, the second *p*-value for the Serial test, the backward direction for the Cumulative Sums test, the +4 state for the Random Excursions test, and the +9 state for the Random Excursions Variant test. More detailed reporting information, test parameters, stream-level results, and all sub-results for tests with multiple outputs are provided in Appendix C, Table A4.

As shown in Table 4, all representative *p*-values reported for R1, R1-ext, R2, and R3 satisfied the *p* ≥ 0.01 criterion, and a 10/10 pass count was obtained for the reported output of each test. The absence of any reported failure in the R1-ext setting indicates that the proposed generation pipeline preserved acceptable bit-level statistical behavior under an independent real genomic data source. Similarly, the R3 setting, which does not include any training or optimization step, also passed the reported tests. This suggests that the observed bit-level behavior is not solely attributable to patterns learned from training data.

Because this evaluation is limited to 10 independent streams, the results should not be interpreted as a substitute for the second-level *p*-value uniformity or pass-rate analysis of NIST SP 800-22, which requires larger sample sets. Instead, the findings are interpreted as stream-level pass results and descriptive statistical summaries. Overall, these results indicate that the bitstreams generated without an external post-processing layer satisfy the NIST SP 800-22 stream-level pass criterion reported in Table 4. However, these results alone should not be regarded as a formal proof of cryptographic security.

#### 4.2.2. SP 800-90B-Inspired Min-Entropy, Independent and Identically Distributed (IID)-Related Indicators, and Health-Test Evaluation

The statistical properties of the bitstreams generated by the proposed DNA-local PRNG framework were further evaluated using SP 800-90B-inspired lower-bound min-entropy estimators, IID-related indicators, and online health-test measures [18,19]. This analysis was included because an evaluation based only on bit balance or a single entropy measure may remain limited. Such measures may not fully capture dominance of the most likely output or short-range dependencies. Min-entropy estimators examine output unpredictability from a conservative lower-bound perspective. Using complementary estimators also reduces dependence on a single modeling assumption. For each of the R1, R1-ext, R2, and R3 settings, 10 independent 1 Mbit bitstreams were analyzed under the same evaluation protocol.

The evaluation used the most common value (MCV), t-tuple, and first-order Markov min-entropy estimators. In addition, collision-based H_2_ and compression-based auxiliary lower-bound indicators were reported for comparison. To examine bit balance and low-order dependence, the 1-bit proportion p(1), Monobit and Runs *p*-values, and first-order mutual information were reported. Time-local anomalies were evaluated using the Repetition Count Test (RCT) and Adaptive Proportion Test (APT). Table 5 presents the mean ± sample SD and Student-t-based 95% CIs calculated from 10 independent streams. Stream-level detailed results are provided in Appendix D, Table A5.

As shown in Table 5, p(1) values remained close to 0.5 in all settings. According to the more conservative t-tuple estimator, regime-averaged min-entropy lower bounds ranged from 0.9955 to 0.9964 bit/bit. The MCV and first-order Markov estimators produced values close to 1 bit/bit. The collision- and compression-based auxiliary lower-bound indicators were also consistent with this overall pattern. The R1-ext setting showed a descriptive profile similar to R1. This indicates that no clear descriptive degradation was observed when the generation pipeline was evaluated under an independent real genomic source. The R3 setting, despite having no training or optimization step, produced min-entropy values at a similar magnitude to the trained settings. This suggests that the observed lower-bound unpredictability behavior is not solely attributable to patterns learned from training data.

The IID-related indicators support the same interpretation. In the stream-level results reported in Appendix D, the Monobit and Runs *p*-values remained above the 0.01 threshold for all streams. First-order mutual information values were on the order of 10^−7^–10^−6^ bit. The maximum RCT run lengths remained clearly below the predefined 40-bit threshold, and all RCT and APT checks were within the acceptance ranges. The APT worst-window *p*-values were reported as descriptive indicators of local fluctuations and were not interpreted as standalone failure decisions.

Overall, these findings show that the bitstreams generated under the four settings exhibited high lower-bound min-entropy, weak first-order dependence, and acceptable health-test behavior. However, the results were not used to establish a statistical superiority ranking among regimes. They were interpreted as descriptive reliability indicators. In addition, this evaluation should not be regarded as formal SP 800-90B entropy certification, complete IID validation, or a formal proof of cryptographic security.

#### 4.2.3. ENT Tests

The bitstreams generated from the proposed DNA-local PRNG framework were also evaluated using the ENT-style statistical measures implemented in Python in the manuscript [20,21]. This analysis was conducted to examine the classical statistical properties and information density of the bitstreams from a complementary perspective. ENT reports byte-level summary measures, including entropy per byte, the chi-square statistic, arithmetic mean, Monte Carlo π estimate, and serial correlation coefficient between consecutive bytes. Therefore, ENT results were not interpreted as a replacement for the NIST SP 800-22 or SP 800-90B-inspired evaluations. Instead, they were treated as a complementary and descriptive assessment of the classical statistical behavior of the bitstreams. For each of the R1, R1-ext, R2, and R3 settings, 10 independent 1 Mbit bitstreams were analyzed under the same ENT evaluation protocol. Table 6 presents the mean ± sample SD and Student-t-based 95% CIs calculated for each setting. Stream-level detailed ENT results are provided in Appendix E, Table A6.

As shown in Table 6, the entropy values remained at approximately 7.9985 bits/byte across all settings and were close to the theoretical upper limit of 8 bits/byte. The arithmetic mean values clustered around the expected central value of 127.5, and the 95% CIs of all four settings included this value. These findings indicate that no clear byte-level imbalance or information-density loss was observed in the analyzed bitstreams.

The regime-averaged chi-square statistics were close to the expected 255 degrees of freedom for 256 possible byte values. However, no formal pass decision was made based only on the magnitude of the chi-square statistic. This measure was used descriptively to compare frequency-distribution behavior across regimes. The mean Monte Carlo π estimates remained close to the true value of π, and all 95% CIs included this value. Similarly, the serial correlation coefficients showed small positive or negative values close to zero, and the CIs of all four settings included zero. These results provide complementary evidence that no clear linear dependence was observed between consecutive bytes.

The R1-ext setting showed an ENT profile similar to R1. This indicates that no clear descriptive degradation was observed under an independent real genomic data source. The R3 setting, despite having no training or optimization step, produced ENT results at a similar level to the trained settings. This suggests that the observed ENT profile is not solely attributable to patterns learned from training data.

Overall, the ENT results show that the bitstreams generated under all four settings exhibited high information density, byte distributions close to expected central values, and negligible serial correlation. However, these results were not used to establish a statistical superiority ranking among regimes and should not be interpreted as a standalone formal proof of cryptographic security.

### 4.3. DNA-Specific Structural and Multi-Stream Evaluation

#### 4.3.1. DNA-Specific Metrics

The structural compatibility of the proposed DNA-local PRNG framework was evaluated using DNA-channel-specific metrics in addition to bit-level randomness tests. For this purpose, the 10 independent 500,000-base DNA streams generated under each of the R1, R1-ext, R2, and R3 settings were analyzed in terms of GC ratio, maximum homopolymer length, normalized Lempel–Ziv (LZ) complexity, 3-mer distribution behavior, reverse-complement (RC) symmetry, block entropy, bit-level p(1), and zlib compression ratio. Thus, the evaluation covered not only the classical randomness properties of the derived bitstreams, but also structural features that may be relevant for DNA-based representation and potential synthesis/sequencing workflows. Table 7 presents the mean ± sample SD and Student-t-based 95% CIs calculated from 10 independent streams for each setting. Stream-level detailed metric results are provided in Appendix F, Table A7.

As shown in Table 7, the GC ratio remained close to the target value of 0.5 in all settings, and the maximum homopolymer length did not exceed the design limit of 5 in any independent stream. These results indicate that the GC-balancing and homopolymer-control mechanisms defined in the constraint-aware sampling layer were preserved over long DNA streams. The same constraints were also maintained in the R1-ext setting, suggesting that this behavior was retained under an independent real genomic source.

The block entropy results show that H(1)–H(6) values remained close to their corresponding theoretical upper bounds across all settings. This supports the interpretation that the generated DNA sequences exhibited high symbolic diversity over the four-symbol alphabet. Normalized LZ complexity values were also high in all settings. These values were approximately 0.986 in R2 and R3, whereas R1 and R1-ext showed slightly lower but still high complexity profiles. This difference suggests that synthetic or reference-free generation settings may exhibit a more isotropic symbolic profile, while real-data-related settings may partially retain short-range source-dependent structural traces.

The very small 3-mer *p*-values indicate that local 3-mer distributions cannot be fully explained by an equiprobable and memoryless four-symbol source. However, the aim of the proposed framework is not to make all k-mer distributions completely uniform. Rather, it aims to generate controlled and reproducible sequences under DNA-compatibility constraints. Therefore, the 3-mer findings were not interpreted as standalone failure indicators. They were treated as descriptive indicators of local motif and transition structures.

The RC-symmetry results also reveal regime-dependent structural differences. The R2 and especially R3 settings showed more balanced RC-symmetry *p*-value profiles than R1 and R1-ext. In the real-data-related R1 and R1-ext settings, smaller *p*-values were observed at the k = 2 and k = 3 levels. This suggests that short-range RC-related structural traces were not fully suppressed in real-data-related generation, but were reflected in the generation pipeline in a controlled manner. This observation supports the interpretation that the method is not merely an abstract randomness generator. Rather, it provides a production architecture that makes the interaction between the data source and constraint-aware sampling visible at the DNA level.

The bit-level p(1) values remained close to 0.5 in all settings. The zlib compression ratios were also approximately 0.1498 across all four settings and did not show a clear regime-level separation. This metric was interpreted as an auxiliary compressibility indicator that is sensitive to output representation and local repeat structure. It was not used as a direct measure of superiority or failure. Overall, the DNA-specific metrics show that the proposed generation pipeline preserved core DNA-compatibility targets, including GC balance and the homopolymer limit. At the same time, it was able to reflect regime-dependent local structural traces in a controlled manner. These results provide supporting evidence for structural compatibility and generation behavior, but they should not be interpreted as a formal proof of cryptographic security.

#### 4.3.2. Multi-Stream Independence and Leakage Controls

To evaluate whether the proposed DNA-local PRNG framework behaves independently not only at the single-stream level but also across different generation runs, two complementary analyses were performed. First, in the data-supported settings, an exact-match k-mer-based leakage control was applied to examine whether the generated DNA streams directly copied long fragments from the corresponding reference or training corpora. Second, all pairwise comparisons were performed among the 10 independent streams generated with different run_seed values in each regime. The Hamming ratio was calculated in the bit domain, and the mismatch ratio was calculated in the DNA domain.

The leakage analysis was applied to the R1, R1-ext, and R2 settings, because these settings are associated with real genomic, independent real genomic, and synthetic reference/training corpora, respectively. In each setting, a 1,000,000-base reference or source DNA sequence was compared with 10 independently generated 500,000-base DNA streams. For k = 32, 48, and 64, the direct k-mer matching control showed no long k-mer overlap with the reference or training corpus in R1, R1-ext, or R2. The R3 setting was not included in this analysis because it does not use any external training or reference corpus. Table 8 summarizes the exact-match k-mer leakage control results.

For multi-stream independence analysis, all pairwise stream combinations were formed from the 10 independent streams in each regime:$\;\left(\begin{array}{c} 10\\ 2 \end{array}\right)=45$. Thus, 45 stream pairs were evaluated for each regime. In the bit domain, the expected Hamming ratio for two independent and balanced bitstreams was defined as:$\;p_{0}=0.5$. In the DNA domain, the expected mismatch ratio under a four-symbol uniform and independent source was defined as: $p_{0}=1-\sum_{b\in \Sigma }P{\left(b\right)}^{2}=0.75.$ In this framework, Hamming ratios close to 0.5 support independence between bitstreams, whereas mismatch ratios close to 0.75 are consistent with four-symbol uniform-independent behavior in the DNA domain. Table 9 presents the pooled Hamming and mismatch results calculated over 45 stream pairs for each regime.

As shown in Table 9, the pooled Hamming ratios in the bit domain remained very close to the theoretical expectation of 0.5 in all regimes. The *p*-values were 0.643, 0.864, 0.292, and 0.471 for R1, R1-ext, R2, and R3, respectively. No significant deviation from the 0.5 expectation was observed in the bit domain. This result supports the absence of systematic inter-stream dependence among bitstreams generated with different seed values.

The DNA-domain results showed a more differentiated pattern. The pooled mismatch ratios in R2 and R3 were 0.749915 and 0.750116, respectively, and were consistent with the theoretical expectation of 0.75. In contrast, the mismatch ratios in R1 and R1-ext were 0.732184 and 0.740847, respectively, and deviated significantly from 0.75. This indicates that, although bit-domain independence was preserved, real-data-related settings partially retained structural traces associated with base distribution and short-range organization in the DNA domain. This result should not be interpreted as a failure. Rather, it is a descriptive finding that makes the DNA-level effect of the data source visible.

To evaluate whether the rule-selection behavior collapsed to a single DNA-to-bit mapping rule, normalized rule entropy was calculated from the usage distribution of the eight rules in each run. Under ideal balanced usage, this value approaches 1. The normalized rule entropy values for R1, R1-ext, R2, and R3 were 0.9999965 ± 0.0000015, 0.9999961 ± 0.0000011, 0.9999960 ± 0.0000016, and 0.9999977 ± 0.0000011, respectively. The mean dominant-rule ratio remained approximately within the range of 0.1256–0.1258 across all settings. These results show that adaptive DNA-to-bit rule selection did not collapse to a single rule and that the bitstream did not become overly dependent on a fixed mapping rule. Rule entropy is not interpreted as a cryptographic security proof. It is used as a descriptive indicator supporting the interpretability of rule-selection behavior. A detailed rule-usage summary is provided in Appendix G, Table A8, and run-level rule traces and raw outputs are shared in the GitHub repository for reproducibility.

Overall, the leakage and multi-stream independence analyses indicate that the proposed method did not show evidence of direct long-fragment reproduction from reference or training sequences. In the DNA domain, especially in the real-data-related R1 and R1-ext settings, source-sensitive structural traces were partially preserved. This suggests that the method is not merely an abstract bitstream generator. Instead, it provides a generation architecture that reflects the interaction among DNA-local production, data regime, and constraint-aware sampling. Nevertheless, these analyses provide supporting evidence only for inter-stream independence and direct long-fragment copying control. They do not replace broader attack models or formal security proofs.

#### 4.3.3. Component-Level Sensitivity and Failure-Oriented Stress Control

A limited component-sensitivity and failure-oriented stress control was conducted to evaluate the functional contribution of the generation-time components in the proposed R3 generation pipeline. This analysis was not designed as a full ablation study. Instead, it aimed to examine how DNA-level and bit-level behavior changes when key generation components are removed or restricted. Because R3 does not involve any training data or optimization step, this control was performed under the R3 setting. Thus, the observed changes could be linked more directly to generation-time components related to sampling, constraint management, and DNA-to-bit rule selection rather than to training dynamics.

Four stress variants were evaluated: the fixed-rule variant, in which a single fixed DNA-to-bit rule was used; the no-homopolymer-mask variant, in which the homopolymer mask was removed; the no-lag1-suppression variant, in which lag-1 suppression was disabled; and the no-constraint variant, in which GC balancing, the homopolymer mask, and lag-1 suppression were jointly disabled. Each variant was run with three independent seed values. In each run, a 500,000-base DNA sequence and the corresponding 1,000,000-bit stream were generated. The summary evaluation was based on GC ratio, maximum homopolymer length, bit-level proportion of ones, Monobit behavior, and normalized rule entropy. Table 10 presents the comparative summary of the evaluated methods and highlights the main distinctions of the proposed approach. Run-level detailed results are provided in Appendix H, Table A9 and Table A10.

The fixed-rule variant clearly demonstrated the effect of dynamic DNA-to-bit rule selection on bit-level balance. In this variant, the GC ratio remained within the target range, the maximum homopolymer length did not exceed the predefined limit, and DNA symbol entropy remained high. In contrast, the bit p(1) value systematically deviated from 0.5, and the Monobit *p*-values remained far below the acceptable range. This finding shows that DNA-level structural compatibility alone does not guarantee bit-level balance. Therefore, dynamic rule selection should be regarded not merely as an additional coding preference, but as a functional component that supports the balance of the corresponding bitstream.

The no-homopolymer-mask variant showed the opposite behavior. In this variant, bit p(1), Monobit behavior, and rule-usage balance were largely preserved. However, the maximum homopolymer length increased to the range of 16–20 across the three independent runs. Similarly, in the no-constraint variant, the maximum homopolymer length increased to the range of 19–23, and a limited decrease in DNA symbol entropy was observed. These findings indicate that an output that appears balanced at the bit level may still be structurally unsuitable at the DNA level. Therefore, the homopolymer mask and the in-generation constraint layer are necessary functional components for DNA-local generation.

In the no-lag1-suppression variant, no clear degradation was observed in terms of GC balance, homopolymer limit, bit p(1), or rule entropy. This suggests that lag-1 suppression does not have as dominant an effect on the primary DNA-compatibility metrics as the homopolymer mask or the general constraint layer. However, this does not imply that lag-1 control is unnecessary. Rather, it indicates that lag-1 suppression should be interpreted as a supporting regulatory component that may be more relevant for detailed analyses of short-range dependencies and local motif behavior.

Overall, this limited stress control shows that the components in the proposed generation pipeline contribute functionally at different levels. Dynamic DNA-to-bit rule selection supports the preservation of bit-level balance, whereas the homopolymer mask and the in-generation constraint layer play a decisive role in preserving DNA-level structural compatibility. This analysis should not be interpreted as a formal security proof or a full ablation study. Nevertheless, by showing failure-like behavior when specific components are removed, it experimentally supports the design rationale of the proposed DNA-local PRNG core.

### 4.4. Performance and Computational Complexity Analysis

#### 4.4.1. Empirical Performance Analysis

The operational behavior of the proposed DNA-local PRNG core was evaluated over 10 independent streams under the R1, R1-ext, R2, and R3 settings. In each run, a 500,000-base DNA sequence was generated, and the corresponding 1,000,000-bit stream was obtained. The performance evaluation used four main measures: wall-clock execution time, cumulative central processing unit (CPU) time, additional memory usage relative to the initial state, and generation efficiency. Efficiency was calculated as the ratio of the number of generated DNA bases to the total execution time and was reported in bases/minute.

To support comparability, all measurements were obtained under CPU-only execution conditions in the same hardware and software environment. For R1, R1-ext, and R2, the reported total time and CPU load represent the combined cost of the single-epoch training stage and the subsequent generation stage. In contrast, because the R3 setting does not include any training or optimization stage, only the generation process was measured. Therefore, the results should not be interpreted as a comparison of pure inference speed alone. They represent the total operational cost of each regime under its own experimental definition. Table 11 presents the mean ± sample SD and Student-t-based 95% CIs calculated over 10 independent runs for each regime. Run-level detailed performance records are provided in Appendix B, Table A3.

As shown in Table 11, the mean execution times ranged from 26.34 to 31.37 min across regimes. The R3 setting, which does not include a training stage, showed the shortest total execution time. In the training-supported regimes, the total times reflect the combined effect of training and generation. The higher mean execution time observed for R1-ext compared with R1 and R2 indicates the additional operational cost of training-supported generation under an independent real genomic source. The CPU-time results showed a similar pattern. The mean CPU time was approximately 157.16 min for R3, compared with 167.84 min for R1, 180.37 min for R2, and 186.29 min for R1-ext. These results indicate that the training-supported settings required higher CPU load. Nevertheless, all regimes operated within the same order of magnitude in terms of operational cost. Additional memory usage remained approximately 16–17 MB for R1 and R2. The corresponding values were 23.50 MB for R1-ext and 31.73 MB for R3. The higher memory usage in R3 suggests that the generation-only execution profile may differ from the training-supported settings in terms of memory behavior. However, the observed increase was limited and did not reach a level that would prevent execution under CPU-only conditions. Generation efficiency was highest in R3, reaching approximately 19,002 bases/minute. The mean efficiencies for R1, R2, and R1-ext were approximately 17,823, 16,585, and 15,953 bases/minute, respectively. This difference is consistent with the absence of training cost in R3. Although the training-supported settings showed lower efficiency, their total time, CPU load, and memory usage remained within practical execution limits.

Overall, the performance results indicate that the proposed DNA-local PRNG framework provides an applicable time–resource balance under the R1, R1-ext, R2, and R3 settings. R3 achieved shorter execution time and higher generation efficiency, whereas R1, R1-ext, and R2 showed acceptable CPU-only execution profiles despite the additional cost of the training stage. These findings support that the method can operate at the experimental scale with a feasible computational cost, in addition to satisfying the statistical and DNA-specific evaluation criteria.

#### 4.4.2. Theoretical Computational Complexity

The theoretical computational cost of the proposed decoder-only Transformer-based DNA-local PRNG core was analyzed separately for the generation and training stages. The generation complexity applies to all R1, R1-ext, R2, and R3 settings, because all regimes use the same basic autoregressive generation core. Training complexity was evaluated only for the data-supported R1, R1-ext, and R2 settings. Since R3 does not include any training or optimization stage, only generation cost was considered for this regime.

In the analysis, the model is assumed to be a decoder-only Transformer core with L layers, h attention heads, and embedding dimension d. The context-window length is denoted by W, the generated sequence length by G, the batch size by B, and the total number of training tokens by P. In the configuration used in this study, L=3, h=4, d=128, and W=128. The four-class base head and the eight-class rule head are treated as low-order output operations that do not change the dominant asymptotic terms. During generation, the use of a KV cache and a fixed-length sliding window avoids recomputing the entire past sequence for each new token. Therefore, the dominant cost for each new token consists of attention computation over the window and the feed-forward projection. The first W tokens introduce a one-time initialization cost. Accordingly, the total generation time can be expressed as Equation (22):

(22) $$T_{gen}=\Theta \left(L\left(W^{2}d+Wd^{2}\right)\right)+\;\Theta \left(GL\left(Wd+d^{2}\right)\right)$$

The first term in Equation (22) represents the initialization of the starting context window. The second term represents the main generation stage of length G. In the typical case where G≫W, and L, W, and d are fixed, the generation cost grows approximately linearly with the output length G. This property demonstrates the main scalability advantage of the sliding-window and KV-cache design when generating long DNA sequences and the corresponding bitstreams. Generation memory mainly consists of model parameters and KV-cache components. Therefore, generation memory can be approximated as Equation (23):

(23) $${Mem}_{gen}=\Theta \left(Ld^{2}\right)+\Theta \left(BLWd\right)$$

Here, the first term represents model parameters, whereas the second term represents the KV-cache cost determined by batch size, number of layers, window length, and embedding dimension. When a fixed window length is used, generation memory does not grow with the total output length G. Instead, it is mainly determined by L, W, d, and B. This keeps memory requirements controlled during long-stream generation. During training, attention is computed over the full window at each training step. Therefore, the single-step cost is higher than the per-token generation cost. For a single training step, the dominant forward-pass cost is given in Equation (24):

(24) $$T_{step}=\Theta \left(BL\left(W^{2}d+Wd^{2}\right)\right),$$

For single-epoch training, the approximate number of steps is: $\left(N_{step}\right)$ $\approx \frac{P}{B\;W}$. Thus, the total training time can be expressed as Equation (25):

(25) $$T_{train}=\Theta \left(LP\left(Wd+d^{2}\right)\right).$$

Although Equation (25) shows that the training cost increases with the total number of training tokens P, context-window length W, embedding dimension d, and number of layers L. Optimization methods such as AdamW may introduce additional overhead through parameter updates and optimizer-state memory. However, they do not change the dominant time-order term. This explains why the R1, R1-ext, and R2 settings showed higher CPU time than R3 in the empirical results. Training memory consists of model parameters, optimizer states, and activations. Let κ denote the relative contribution of optimizer-state memory compared with parameter memory. Training memory can be approximated as Equation (26):

(26) $${Mem}_{train}=\Theta ((1+\kappa )Ld^{2})+\left\{\begin{array}{cc} \Theta (BL{(Wd+hW}^{2}))\; & classical\;attention\;implementation\\ \Theta (BLWd)\; & SDPA-compatible\;attention\;implementation \end{array}\right.$$

In a classical attention implementation, storing attention weights explicitly may introduce an additional activation-memory term of order ${hW}^{2}$. In Scaled Dot-Product Attention (SDPA)-compatible implementations, this burden is reduced, and practical memory behavior is more closely associated with the BLWd term. Nevertheless, training memory remains higher than generation memory because activations must be stored for backpropagation.

In this study, the 500,000-base DNA output and the corresponding 1,000,000-bit stream length were selected as the main experimental scale because they are sufficient for bit-level randomness tests and DNA-specific structural evaluation. Longer DNA and bitstreams can be generated using the same pipeline. However, the primary aim of this study was to characterize the proposed core under standard randomness tests, DNA-specific metrics, and reproducible multi-run settings. Owing to the fixed context window and KV-cache mechanism, the generation cost is expected to scale approximately linearly with output length when model size and window length are fixed. Therefore, larger-scale experiments can be addressed in future work without changing the basic design of the method.

Overall, the theoretical analysis shows that the proposed core scales approximately linearly with output length during generation, while generation memory depends on the model and window dimensions rather than the total generated length. The training stage is more costly because of full-window attention computation and activation memory. Nevertheless, the lightweight configuration used in this study provides a controlled balance between methodological adequacy and computational feasibility. These results are consistent with the empirical performance findings reported in Section 4.4.1 and support the computational applicability of the proposed DNA-local PRNG core for long-stream generation.

## 5. Discussion

When the findings of this study are considered together with selected machine-learning-based PRNG literature, the proposed DNA-local Transformer-based framework differs in several important respects. As summarized in Table 12, Transformer-based [8], LSTM-based [22,25], GAN/WGAN-based [26], and reinforcement-learning-based [30] studies generally focus on bit-domain generation, and their evaluations are often based on a limited number of statistical tests. In contrast, the outputs generated in this study were evaluated not only using NIST SP 800-22, but also through SP 800-90B-inspired min-entropy and health indicators, ENT statistics, DNA-specific structural metrics, multi-stream independence, exact-match leakage controls, empirical performance, and theoretical complexity. This multilayer protocol was not used to claim formal cryptographic security. Rather, it was used to characterize the statistical, structural, and operational behavior of the proposed generator more transparently.

One of the main distinctions of the proposed method is that generation is not first performed in the bit domain and then converted into DNA symbols. Instead, generation is carried out directly in the A/C/G/T alphabet, while GC balance, homopolymer limitation, short-range dependency suppression, and local n-gram behavior are managed during generation [3,4,5,6]. The results show that this choice is not only methodological, but also operationally meaningful. The GC ratio remained close to the target value, the maximum homopolymer length did not exceed the predefined limit, and a controlled structural profile was preserved over long 500,000-base DNA streams. Therefore, the proposed method should not be interpreted merely as a bitstream generator with an added DNA representation layer. It should instead be regarded as a DNA-local generation architecture in which production constraints directly shape sequence formation.

The transition from the DNA domain to the bit domain was also handled differently from fixed-mapping approaches. In the proposed framework, the bitstream is derived not through a single fixed DNA-to-bit conversion rule, but through dynamic selection among eight equivalent coding rules. This design aims to reduce secondary regularities that may arise when a single mapping rule is used throughout the entire sequence. The consistency of the NIST SP 800-22, SP 800-90B-inspired, and ENT results suggests that dynamic rule selection may contribute to bit-domain behavior. However, this interpretation should be treated cautiously. It is not a security proof or a standalone ablation result, but an interpretation supported by the experimental findings.

Another important finding is that the effect of the data regime becomes visible in the DNA domain. Evaluating R1, R2, and R3 under a common protocol made it possible to examine how the observed behavior relates to real-data, synthetic-data, and reference-free generation conditions. In the bit domain, all regimes showed strong statistical profiles and multi-stream independence. In the DNA domain, however, the regimes exhibited more distinguishable structural properties. The significant deviation of the pooled mismatch ratio from the theoretical expectation of 0.75 in R1 and R1-ext suggests that short-range DNA-structural traces are partially preserved in real-data-related generation. This should not be interpreted as a failure. Rather, it is a descriptive finding that makes the DNA-level effect of the data source visible. By contrast, R2 and especially R3 showed more isotropic DNA-level behavior.

The leakage analysis complements this interpretation. In the trained or reference-associated settings, no exact-match long k-mer overlap was observed between the generated DNA streams and the corresponding reference/training corpus for k = 32, 48, and 64. This finding indicates that, under the tested criteria, long reference fragments were not directly copied. However, the absence of exact-match hits should not be interpreted as formal proof that all possible forms of memorization are absent. As indicated by the DNA-specific structural metrics, local structural traces related to the data regime can still be reflected in the generation pipeline in a controlled manner, even without direct copying.

The empirical performance and theoretical complexity findings also support the applicability of the method. As expected, R1, R1-ext, and R2 required higher total execution time and CPU load because they included a training stage. In contrast, R3 consisted only of the generation stage and therefore achieved shorter execution time and higher generation efficiency. Nevertheless, the computational costs of all regimes remained within the same order of magnitude. The theoretical analysis is consistent with this observation. Because of KV-cache and sliding-window operation, generation cost scales approximately linearly with output length, while generation memory depends mainly on the model and window dimensions rather than the total generated length. This indicates that the proposed DNA-local PRNG core provides a computationally feasible profile at the evaluated experimental scale.

The proposed framework may be useful in several DNA-oriented computational workflows where randomness must be considered together with sequence-level structural constraints. Potential application areas include in silico DNA sequence design, DNA data storage and indexing, generation of structurally constrained synthetic DNA streams for benchmarking, and simulation of DNA-based processing pipelines. Because the generator operates directly in the A/C/G/T alphabet, it can produce candidate sequences while monitoring GC balance, homopolymer length, local motif behavior, and the corresponding bitstream within the same pipeline. This feature may be particularly useful when DNA-compatible random or pseudorandom sequences are required before downstream synthesis, sequencing, storage, or computational evaluation. However, the proposed framework should not be interpreted as a replacement for conventional CSPRNGs or as a formally secure cryptographic primitive; its practical relevance lies primarily in DNA-local, constraint-aware, and reproducible stream generation.

Several limitations of this study should also be stated clearly. First, although the reported findings provide supporting evidence in terms of statistical behavior, structural compatibility, leakage control, and performance, they do not constitute formal CSPRNG-level security proof. Attack models such as state recovery, chosen-seed attacks, internal-state inference, and long-horizon predictability remain outside the scope of this work. Second, the proposed architecture was not directly compared with classical software PRNGs under identical experimental conditions. Therefore, this study does not claim to replace general-purpose PRNGs. Third, although the 1 Mbit bitstream and 500,000-base DNA-stream scale provide a meaningful experimental evaluation, larger-scale experiments under different hardware conditions could further strengthen the conclusions.

In addition, this study did not perform a separate robustness analysis under random base substitutions, noisy variants, or systematically corrupted input sub-corpora. This is because the focus of the study was not to test model robustness under noisy data, but to characterize the statistical, structural, and operational behavior of a constraint-aware and reproducible PRNG core that operates directly in the DNA alphabet. Nevertheless, systematic stress tests under low- and moderate-level base substitutions, motif disruption, or regional composition shifts would be important for future work to assess data-source sensitivity and generation stability in greater detail. Finally, the real-data-related regimes were evaluated using a limited number of genomic sources. Therefore, DNA-level structural effects should be interpreted as source-dependent experimental findings rather than as a complete characterization across all genomic distributions.

Overall, this study positions the Transformer architecture not merely as a general-purpose generator operating on bit sequences, but as a DNA-local PRNG core capable of sampling directly in the DNA alphabet under explicit generation-time constraints. Evaluating the same architectural core under real-data, synthetic-data, and reference-free generation conditions within a common protocol shows that the method provides a multidimensional and analyzable framework that is not tied to a single data regime. When considered together with the selected literature and the findings reported in this study, the proposed approach addresses a meaningful gap in machine-learning-based PRNG research in terms of DNA-local randomness generation, in-generation structural constraint control, and multilayer validation.

## 6. Conclusions

This study presented a constraint-aware, reproducible, Transformer-based DNA-local PRNG framework for DNA-oriented random sequence generation. Unlike approaches that first generate outputs in the bit domain and then map them to DNA symbols, the proposed method performs generation directly in the A/C/G/T alphabet. This structure allows GC balance, homopolymer limits, and short-range sequence regularities to be managed during generation, while the corresponding bitstream is derived through dynamic selection among eight equivalent DNA-to-bit coding rules. The proposed core was evaluated under a common validation protocol across R1 associated with real genomic data, R1-ext based on an independent real genomic source, R2 associated with synthetic data, and R3 without training or reference data. For each setting, 10 independent generation runs were performed, and the results were reported with mean, sample standard deviation, 95% confidence intervals, and stream-level details. Bit-level evaluations using NIST SP 800-22, SP 800-90B-inspired min-entropy and health indicators, and ENT showed a stable statistical profile. DNA-specific analyses showed that the GC ratio remained close to the target value, the homopolymer limit was preserved, and the generated sequences exhibited high symbolic diversity. Multi-stream independence and exact-match k-mer leakage controls did not indicate systematic bit-level dependence under different seed values or direct long-fragment copying from reference/training corpora. The performance and theoretical complexity analyses showed that the proposed framework provides an applicable time–resource balance at the experimental scale. The training-free R3 setting achieved shorter execution time and higher generation efficiency, whereas R1, R1-ext, and R2 exhibited acceptable CPU-only execution profiles despite the additional training cost. The theoretical analysis further supported that, with KV-cache and a fixed-length sliding window, generation cost scales approximately linearly with output length. Nevertheless, this study does not provide a formal CSPRNG-level cryptographic security proof. Attack models such as state recovery, chosen-seed attacks, internal-state inference, and long-horizon predictability remain outside the scope of this work. Future studies should examine longer streams, more diverse genomic sources, different model and window configurations, fixed-rule ablations, separate removal of constraint components, and explicit attack models. Overall, the findings indicate that the proposed method addresses a meaningful gap in machine-learning-based PRNG research through DNA-local generation, in-generation constraint control, dynamic DNA-to-bit mapping, and multilayer validation.

## 7. Patents

The work reported in this manuscript has resulted in a national patent application titled “Eğitim Gerektirmeyen, Biyolojik Kısıt Farkındalıklı Transformer Tabanlı DNA Sözde Rastgele Dizi Üretimi ve Kodlama” (Training-Free, Biological Constraint-Aware Transformer-Based DNA Pseudorandom Sequence Generation and Encoding). The invention has been officially categorized as a “Service Invention” and approved for filing by the Fırat University Executive Board (Decision No: 2025-2026/17.43, dated 11 March 2026). The application process is currently being managed by the Fırat Technology Transfer Office.

## Figures and Tables

**Table 1:** Comparison of representative PRNG and DNA-coding approaches.

Ref.	Method Class	Output Space	DNA-Local Generation	In- Generation Constraints	Dynamic Rule Selection	Referencefree Generation Setting	Integrated Validation
[8]	Transformer-based PRNG	Bit	No	No	Not applicable	No	No
[11]	Transformer-based PRNG	Bit	No	No	Not applicable	No	No
[22]	LSTM-based PRNG	Bit	No	No	Not applicable	No	No
[24]	GAN-based PRNG	Bit	No	No	Not applicable	No	No
[28]	WGAN-GP-based PRNG	Bit	No	No	Not applicable	No	No
[25]	RL-based PRNG	Bit	No	No	Not applicable	No	No
[30]	RL-based PRNG	Bit	No	No	Not applicable	No	No
[5]	DNA coding-based approach	Bit → DNA	No	Partial	Partial	No	No
[12]	Dynamic DNA coding	Bit → DNA	No	Partial	Yes	No	No
[31]	Hybrid CNN + chaos + DNA	Bit → DNA	No	Partial	Variable	No	Partial
This work	Dual-head Transformer-based PRNG	DNA and corresponding bitstream	Yes	Yes	Yes	Yes	Yes

**Table 2:** Architectural comparison between the original Transformer and the proposed DNA-PRNG core.

Component	Original Transformer [7]	Proposed DNA-PRNG Core
Objective	Sequence-to-sequence modeling/machine translation	DNA-local PRNG and dynamic DNA-to-bit rule selection
Architecture	Encoder–decoder structure with cross-attention	Decoder-only, 3 blocks, no cross-attention
Positional coding	Fixed sinusoidal coding	RoPE on query and key vectors; sliding window and KV cache
Capacity	$d_{model\;}$ = 512, 6 blocks	$d_{model\;}$ = 128, 3 blocks
Number of attention heads	8/16	4
Output heads	Single token head	Dual head: base head (4), rule head (8)
DNA-specific constraints	Not explicitly defined	Applied during generation: GC balance, homopolymer limit, lag-1 damping
Sampling	Task-dependent	Constraint-aware softmax, generation-time masks, and rule-guided selection
Context handling	Task-dependent	KV cache and sliding window (W = 128)
Reproducibility	Not explicitly targeted	Seed-controlled and traceable execution

**Table 3:** Eight equivalent DNA-to-bit coding rules.

Base	Rule-1	Rule-2	Rule-3	Rule-4	Rule-5	Rule-6	Rule-7	Rule-8
A	00	11	00	11	01	10	01	10
C	01	01	10	10	00	00	11	11
G	10	10	01	01	11	11	00	00
T	11	00	11	00	10	01	10	01

**Table 4:** Descriptive summary of NIST SP 800-22 test results for 10 independent 1 Mbit bitstreams under the R1, R1-ext, R2, and R3 settings.

Regime	Test	Mean ± SD	%95 CI	Min–Max	Pass Count
R1	T1 Monobit	0.587668 ± 0.348857	[0.338111, 0.837225]	0.114107–0.980853	10/10
	T2 Block Frequency *	0.560295 ± 0.332859	[0.322182, 0.798408]	0.089008–0.976522	10/10
	T3 Runs	0.646181 ± 0.298660	[0.432532, 0.859830]	0.224240–0.982196	10/10
	T4 Longest Run	0.562453 ± 0.344726	[0.315851, 0.809055]	0.060961–0.997963	10/10
	T5 Rank	0.500286 ± 0.241495	[0.327531, 0.673041]	0.152231–0.898560	10/10
	T6 DFT	0.430075 ± 0.277980	[0.231220, 0.628929]	0.056296–0.832839	10/10
	T7 Non-overlapping Template *	0.482907 ± 0.290107	[0.275377, 0.690436]	0.095713–0.990490	10/10
	T8 Overlapping Template	0.603092 ± 0.283364	[0.400385, 0.805798]	0.094000–0.925462	10/10
	T9 Universal	0.435935 ± 0.294194	[0.225481, 0.646389]	0.047189–0.924031	10/10
	T10 Linear Complexity	0.458431 ± 0.273526	[0.262762, 0.654099]	0.120095–0.958357	10/10
	T11 Serial *	0.465805 ± 0.292134	[0.256826, 0.674785]	0.032145–0.907015	10/10
	T12 Approximate Entropy	0.630826 ± 0.287497	[0.425163, 0.836489]	0.015260–0.950942	10/10
	T13 Cumulative Sums (Backward)	0.558479 ± 0.310912	[0.336065, 0.780892]	0.134201–0.964088	10/10
	T14 Random Excursions *	0.509390 ± 0.277053	[0.311199, 0.707582]	0.087099–0.940605	10/10
	T15 Random Excursions Variant *	0.672413 ± 0.259892	[0.486498, 0.858328]	0.073160–0.967530	10/10
R1-ext	T1 Monobit	0.475082 ± 0.339689	[0.232083, 0.718081]	0.023451–0.918757	10/10
	T2 Block Frequency *	0.460501 ± 0.164257	[0.342999, 0.578003]	0.273553–0.793153	10/10
	T3 Runs	0.453489 ± 0.375930	[0.184565, 0.722413]	0.018546–0.964890	10/10
	T4 Longest Run	0.704891 ± 0.264733	[0.515512, 0.894269]	0.112640–0.990511	10/10
	T5 Rank	0.640300 ± 0.323552	[0.408845, 0.871756]	0.122663–0.988609	10/10
	T6 DFT	0.543136 ± 0.345497	[0.295982, 0.790289]	0.011924–0.948782	10/10
	T7 Non-overlapping Template *	0.507921 ± 0.305625	[0.289290, 0.726552]	0.081419–0.987610	10/10
	T8 Overlapping Template	0.559410 ± 0.222796	[0.400032, 0.718789]	0.254728–0.904803	10/10
	T9 Universal	0.472334 ± 0.337312	[0.231036, 0.713633]	0.064627–0.976729	10/10
	T10 Linear Complexity	0.584641 ± 0.334033	[0.345688, 0.823593]	0.054720–0.975377	10/10
	T11 Serial *	0.471920 ± 0.338848	[0.229522, 0.714317]	0.010475–0.951755	10/10
	T12 Approximate Entropy	0.697044 ± 0.243492	[0.522861, 0.871228]	0.271060–0.985675	10/10
	T13 Cumulative Sums (Backward)	0.423561 ± 0.324081	[0.191727, 0.655394]	0.029456–0.913034	10/10
	T14 Random Excursions *	0.386287 ± 0.363216	[0.126458, 0.646116]	0.012732–0.934651	10/10
	T15 Random Excursions Variant *	0.430059 ± 0.268530	[0.237964, 0.622153]	0.071589–0.929763	10/10
R2	T1 Monobit	0.459768 ± 0.339386	[0.216986, 0.702550]	0.022608–0.998404	10/10
	T2 Block Frequency *	0.739236 ± 0.196982	[0.598323, 0.880149]	0.328885–0.961222	10/10
	T3 Runs	0.538416 ± 0.267094	[0.347348, 0.729484]	0.145936–0.984091	10/10
	T4 Longest Run	0.453163 ± 0.323608	[0.221668, 0.684659]	0.027110–0.966306	10/10
	T5 Rank	0.546251 ± 0.279573	[0.346257, 0.746245]	0.135480–0.971163	10/10
	T6 DFT	0.419401 ± 0.380305	[0.147348, 0.691455]	0.013919–0.948782	10/10
	T7 Non-overlapping Template *	0.568380 ± 0.213353	[0.415757, 0.721004]	0.299097–0.944411	10/10
	T8 Overlapping Template	0.462864 ± 0.376351	[0.193639, 0.732089]	0.017992–0.985753	10/10
	T9 Universal	0.371665 ± 0.246323	[0.195456, 0.547874]	0.040213–0.923409	10/10
	T10 Linear Complexity	0.437758 ± 0.242766	[0.264094, 0.611423]	0.160839–0.896151	10/10
	T11 Serial *	0.491332 ± 0.354187	[0.237962, 0.744702]	0.059399–0.967183	10/10
	T12 Approximate Entropy	0.370544 ± 0.224451	[0.209981, 0.531106]	0.112163–0.892372	10/10
	T13 Cumulative Sums (Backward)	0.538987 ± 0.330027	[0.302900, 0.775074]	0.023874–0.987844	10/10
	T14 Random Excursions *	0.562245 ± 0.301074	[0.346870, 0.777621]	0.077111–0.937194	10/10
	T15 Random Excursions Variant *	0.421920 ± 0.346657	[0.173937, 0.669903]	0.106827–0.991227	10/10
R3	T1 Monobit	0.599651 ± 0.266945	[0.408690, 0.790612]	0.191553–0.926698	10/10
	T2 Block Frequency *	0.598511 ± 0.311566	[0.375631, 0.821392]	0.111155–0.990253	10/10
	T3 Runs	0.689466 ± 0.320417	[0.460253, 0.918678]	0.068821–0.994978	10/10
	T4 Longest Run	0.442675 ± 0.306665	[0.223300, 0.662050]	0.034811–0.871183	10/10
	T5 Rank	0.622377 ± 0.294478	[0.411720, 0.833034]	0.202430–0.919391	10/10
	T6 DFT	0.622720 ± 0.241634	[0.449865, 0.795574]	0.215403–0.978037	10/10
	T7 Non-overlapping Template *	0.612771 ± 0.315853	[0.386824, 0.838719]	0.010210–0.990923	10/10
	T8 Overlapping Template	0.463382 ± 0.339111	[0.220796, 0.705967]	0.026088–0.999010	10/10
	T9 Universal	0.554283 ± 0.301900	[0.338317, 0.770250]	0.058300–0.918352	10/10
	T10 Linear Complexity	0.566379 ± 0.263632	[0.377788, 0.754971]	0.017217–0.966705	10/10
	T11 Serial *	0.544197 ± 0.185944	[0.411180, 0.677213]	0.337268–0.876529	10/10
	T12 Approximate Entropy	0.633706 ± 0.247900	[0.456369, 0.811043]	0.121313–0.972334	10/10
	T13 Cumulative Sums (Backward)	0.602027 ± 0.293621	[0.391983, 0.812070]	0.213052–0.986478	10/10
	T14 Random Excursions *	0.546712 ± 0.321945	[0.316406, 0.777018]	0.037276–0.939207	10/10
	T15 Random Excursions Variant *	0.590771 ± 0.314462	[0.365818, 0.815723]	0.084301–0.936042	10/10

* For tests with multiple parameter-dependent or multiple *p*-value outputs, the reported value corresponds to the predefined compact-summary rule: M = 1000 for Block Frequency, template 000000001 for Non-overlapping Template, the second *p*-value for Serial, the backward direction for Cumulative Sums, the +4 state for Random Excursions, and the +9 state for Random Excursions Variant.

**Table 5:** SP 800-90B-inspired min-entropy, IID-related, and health-test summaries for 10 independent 1 Mbit bitstreams under the R1, R1-ext, R2, and R3 settings.

Regime	Test Group	Metric	Mean ± SD	95% CI
R1	Entropy tests	p(1)	0.5001 ± 0.0004	[0.4998, 0.5004]
		$H_{MCV}$ (bit/bit)	0.9991 ± 0.0008	[0.9985, 0.9997]
		$H_{min}$ (t-tuple, bit/bit)	0.9964 ± 0.0004	[0.9961, 0.9967]
		$H_{Markov(1)}$ (bit/bit)	0.9987 ± 0.0007	[0.9982, 0.9991]
		$H_{Collision\;H_{2}}$ (bit/bit)	0.9999989 ± 0.0000014	[0.9999979, 0.9999999]
		$H_{Compression\;LB}$ (zlib, bit/bit)	1.0000000 ± 0.0000000	[1.0000000, 1.0000000]
	IID and health tests	Monobit p	0.5877 ± 0.3489	[0.3381, 0.8372]
		Runs p	0.6462 ± 0.2987	[0.4325, 0.8598]
		$I(X_{t};X_{t-1})$ [bits]	(3.0 ± 4.8) × 10^−7^	[−4.6 × 10^−8^, 6.5 × 10^−7^]
		RCT max run	20.7000 ± 2.4967	[18.9140, 22.4860]
		APT worst-window p	(8.07 ± 7.57) × 10^−4^	[2.65 × 10^−4^, 1.35 × 10^−3^]
R1-ext	Entropy tests	p(1)	0.5004 ± 0.0004	[0.5002, 0.5007]
		$H_{MCV}$ (bit/bit)	0.9987 ± 0.0010	[0.9980, 0.9995]
		$H_{min}$ (t-tuple, bit/bit)	0.9955 ± 0.0010	[0.9948, 0.9962]
		$H_{Markov(1)}$ (bit/bit)	0.9980 ± 0.0009	[0.9974, 0.9987]
		$H_{Collision\;H_{2}}$ (bit/bit)	0.9999983 ± 0.0000022	[0.9999968, 0.9999998]
		$H_{Compression\;LB}$ (zlib, bit/bit)	1.0000000 ± 0.0000000	[1.0000000, 1.0000000]
	IID and health tests	Monobit p	0.4751 ± 0.3397	[0.2321, 0.7181]
		Runs p	0.4535 ± 0.3759	[0.1846, 0.7224]
		$I(X_{t};X_{t-1})$ [bits]	(1.0 ± 1.2) × 10^−6^	[1.1 × 10−7, 1.9 × 10^−6^]
		RCT max run	19.7 ± 1.7	[18.5, 20.9]
		APT worst-window p	(4.80 ± 6.82) × 10^−4^	[−8.25 × 10^−6^, 9.67 × 10^−4^]
R2	Entropy tests	p(1)	0.5003 ± 0.0005	[0.5000, 0.5007]
		$H_{MCV}$ (bit/bit)	0.9987 ± 0.0011	[0.9979, 0.9994]
		$H_{min}$ (t-tuple, bit/bit)	0.9958 ± 0.0009	[0.9951, 0.9964]
		$H_{Markov(1)}$ (bit/bit)	0.9983 ± 0.0008	[0.9977, 0.9989]
		$H_{Collision\;H_{2}}$ (bit/bit)	0.9999982 ± 0.0000026	[0.9999964, 1.0000000]
		$H_{Compression\;LB}$ (zlib, bit/bit)	1.0000000 ± 0.0000000	[1.0000000, 1.0000000]
	IID and health tests	Monobit p	0.4598 ± 0.3394	[0.2170, 0.7026]
		Runs p	0.5384 ± 0.2671	[0.3473, 0.7295]
		$I(X_{t};X_{t-1})$ [bits]	(4.0 ± 7.0) × 10^−7^	[−1.0 × 10^−7^, 9.0 × 10^−7^]
		RCT max run	20.4000 ± 1.3499	[19.4343, 21.3657]
		APT worst-window p	(6.23 ± 6.20) × 10^−4^	[1.80 × 10^−4^, 1.07 × 10^−3^]
R3	Entropy tests	p(1)	0.5000 ± 0.0004	[0.4997, 0.5002]
		$H_{MCV}$ (bit/bit)	0.9992 ± 0.0006	[0.9987, 0.9996]
		$H_{min}$ (t-tuple, bit/bit)	0.9957 ± 0.0010	[0.9949, 0.9964]
		$H_{Markov(1)}$ (bit/bit)	0.9988 ± 0.0008	[0.9983, 0.9994]
		$H_{CollisionH_{2}}$ (bit/bit)	0.9999993 ± 0.0000008	[0.9999987, 0.9999999]
		$H_{CompressionLB}$ (zlib, bit/bit)	1.0000000 ± 0.0000000	[1.0000000, 1.0000000]
	IID and health tests	Monobit p	0.5997 ± 0.2669	[0.4087, 0.7906]
		Runs p	0.6895 ± 0.3204	[0.4603, 0.9187]
		$I(X_{t};X_{t-1})$ [bits]	(3.0 ± 6.7) × 10^−7^	[−1.8 × 10^−7^, 7.8 × 10^−7^]
		RCT max run	20.5000 ± 2.7588	[18.5265, 22.4735]
		APT worst-window p	(5.44 ± 4.91 × 10^−4^	[1.93 × 10^−4^, 8.95 × 10^−4^]

Note: Values are reported as the mean ± sample SD and Student-t-based 95% CI calculated over 10 independent bitstreams for each setting. Symmetric CI calculations may produce lower bounds outside the natural range for metrics that are theoretically non-negative or bounded; such limits should be interpreted within the physical range of the corresponding metric. APT worst-window *p*-values are reported as descriptive indicators, and all RCT and APT checks remained within the predefined acceptance ranges. This analysis is inspired by SP 800-90B and should not be interpreted as a formal SP 800-90B entropy certification, a complete IID validation, or a formal proof of cryptographic security.

**Table 6:** Descriptive summary of ENT results for 10 independent 1 Mbit bitstreams under the R1, R1-ext, R2, and R3 settings.

Regime	Metric	Ideal	Mean ± SD	95% CI
R1	Entropy (bits per byte)	≈8.000000	7.998543 ± 0.000131	[7.998449, 7.998637]
	Chi-square statistic	≈255.000	252.366 ± 22.251	[236.448, 268.283]
	Arithmetic mean	≈127.5000	127.5602 ± 0.2119	[127.4086, 127.7118]
	Monte Carlo π estimate	≈3.14159	3.14291 ± 0.00861	[3.13675, 3.14908]
	Serial correlation coefficient	≈0.000000	0.001504 ± 0.002438	[−0.000239, 0.003248]
R1-ext	Entropy (bits per byte)	≈8.000000	7.998517 ± 0.000107	[7.998440, 7.998593]
	Chi-square statistic	≈255.000	256.774 ± 18.521	[243.524, 270.023]
	Arithmetic mean	≈127.5000	127.6446 ± 0.2737	[127.4488, 127.8403]
	Monte Carlo π estimate	≈3.14159	3.13687 ± 0.00984	[3.12983, 3.14391]
	Serial correlation coefficient	≈0.000000	0.000988 ± 0.002444	[−0.000760, 0.002737]
R2	Entropy (bits per byte)	≈8.000000	7.998522 ± 0.000153	[7.998413, 7.998632]
	Chi-square statistic	≈255.000	255.958 ± 26.498	[237.002, 274.914]
	Arithmetic mean	≈127.5000	127.5940 ± 0.1855	[127.4613, 127.7266]
	Monte Carlo π estimate	≈3.14159	3.13976 ± 0.00691	[3.13482, 3.14471]
	Serial correlation coefficient	≈0.000000	−0.000456 ± 0.002881	[−0.002517, 0.001604]
R3	Entropy (bits per byte)	≈8.000000	7.998546 ± 0.000124	[7.998457, 7.998634]
	Chi-square statistic	≈255.000	251.970 ± 21.515	[236.579, 267.360]
	Arithmetic mean	≈127.5000	127.4989 ± 0.1523	[127.3900, 127.6079]
	Monte Carlo π estimate	≈3.14159	3.13952 ± 0.01001	[3.13236, 3.14668]
	Serial correlation coefficient	≈0.000000	−0.000540 ± 0.002951	[−0.002651, 0.001571]

Note: Values are reported as the mean ± sample SD and Student-t-based 95% CI calculated over 10 independent 1 Mbit bitstreams for each setting. Ideal or expected values are provided only for comparison. The chi-square statistic, arithmetic mean, Monte Carlo π estimate, and serial correlation coefficient are interpreted as descriptive indicators. No formal pass or fail decision was made based on these measures. ENT results alone do not constitute a formal proof of randomness or cryptographic security.

**Table 7:** Descriptive summary of DNA-specific structural metrics for 10 independent 500,000-base DNA streams under the R1, R1-ext, R2, and R3 settings.

Regime	Metric	Ideal	Mean ± SD	95% CI
R1	GC ratio	0.50	0.4998 ± 0.0002	[0.4996, 0.4999]
	Homopolymer max	≤5	5.0 ± 0.0	[5.0, 5.0]
	Normalized LZ complexity	≈1.00	0.9664 ± 0.0057	[0.9623, 0.9706]
	$3\text{-mer}\;p\text{-value}\;(\chi^{2}$)	U(0,1)	0.0 ± 0.0	[0.0, 0.0]
	RC-symmetry p (k = 1)	U(0,1)	0.8883 ± 0.1544	[0.7779, 0.9988]
	RC-symmetry p (k = 2)	U(0,1)	1.20 × 10^−4^ ± 3.79 × 10^−4^	[−1.51 × 10^−4^, 3.91 × 10^−4^]
	RC-symmetry p (k = 3)	U(0,1)	1.80 × 10^−18^ ± 5.69 × 10^−18^	[−2.27 × 10^−18^, 5.87 × 10^−18^]
	H(1)	2	2.0000 ± (8.43 × 10^−7^)	[1.9999988, 2.0000000]
	H(2)	4	3.9858 ± 0.0022	[3.9842, 3.9874]
	H(3)	6	5.9659 ± 0.0070	[5.9609, 5.9709]
	H(4)	8	7.9394 ± 0.0146	[7.9289, 7.9498]
	H(5)	10	9.9056 ± 0.0247	[9.8879, 9.9232]
	H(6)	12	11.8586 ± 0.0374	[11.8318, 11.8853]
	Bit p(1)	0.50	0.5001 ± 0.0004	[0.4998, 0.5004]
	zlib ratio	≈0.15	0.1498 ± 0.0001	[0.1498, 0.1499]
R1-ext	GC ratio	0.50	0.4993 ± 0.0003	[0.4991, 0.4995]
	Homopolymer max	≤5	5.0 ± 0.0	[5.0, 5.0]
	Normalized LZ complexity	≈1.00	0.9728 ± 0.0026	[0.9710, 0.9747]
	$3\text{-mer}\;p\text{-value}\;(\chi^{2}$)	U(0,1)	0.0 ± 0.0	[0.0, 0.0]
	RC-symmetry p (k = 1)	U(0,1)	0.3991 ± 0.2420	[0.2260, 0.5722]
	RC-symmetry p (k = 2)	U(0,1)	1.60 × 10^−4^ ± 5.05 × 10^−4^	[−2.01 × 10^−4^, 5.21 × 10^−4^]
	RC-symmetry p (k = 3)	U(0,1)	4.29 × 10^−51^ ± 1.36 × 10^−50^	[−5.42 × 10^−51^, 1.40 × 10^−50^]
	H(1)	2	2.0000 ± (3.75 × 10^−6^)	[1.9999924, 1.9999978]
	H(2)	4	3.9865 ± 0.0019	[3.9851, 3.9878]
	H(3)	6	5.9716 ± 0.0039	[5.9688, 5.9744]
	H(4)	8	7.9549 ± 0.0061	[7.9505, 7.9593]
	H(5)	10	9.9352 ± 0.0089	[9.9288, 9.9415]
	H(6)	12	11.9057 ± 0.0127	[11.8966, 11.9148]
	Bit p(1)	0.50	0.5004 ± 0.0004	[0.5002, 0.5007]
	zlib ratio	≈0.15	0.1498 ± 0.0001	[0.1498, 0.1499]
R2	GC ratio	0.50	0.5000 ± 0.0001	[0.4999, 0.5000]
	Homopolymer max	≤5	5.0 ± 0.0	[5.0, 5.0]
	Normalized LZ complexity	≈1.00	0.9863 ± 0.0005	[0.9860, 0.9866]
	$3\text{-mer}\;p\text{-value}\;(\chi^{2}$)	U(0,1)	1.60 × 10^−8^ ± 5.06 × 10^−8^	[−2.02 × 10^−8^, 5.22 × 10^−8^]
	RC-symmetry p (k = 1)	U(0,1)	0.9952 ± 0.0060	[0.9909, 0.9995]
	RC-symmetry p (k = 2)	U(0,1)	0.0425 ± 0.1288	[−0.0496, 0.1346]
	RC-symmetry p (k = 3)	U(0,1)	0.0156 ± 0.0461	[−0.0174, 0.0486]
	H(1)	2	2.0000 ± 0.0000	[2.0000, 2.0000]
	H(2)	4	3.9997 ± 0.0001	[3.9996, 3.9998]
	H(3)	6	5.9995 ± 0.0003	[5.9993, 5.9996]
	H(4)	8	7.9989 ± 0.0004	[7.9986, 7.9992]
	H(5)	10	9.9974 ± 0.0005	[9.9971, 9.9978]
	H(6)	12	11.9915 ± 0.0006	[11.9911, 11.9920]
	Bit p(1)	0.50	0.5003 ± 0.0005	[0.5000, 0.5007]
	zlib ratio	≈0.15	0.1498 ± 0.0000	[0.1498, 0.1499]
R3	GC ratio	0.50	0.5000 ± 0.0000	[0.5000, 0.5000]
	Homopolymer max	≤5	5.0 ± 0.0	[5.0, 5.0]
	Normalized LZ complexity	≈1.00	0.9864 ± 0.0004	[0.9861, 0.9867]
	$3\text{-mer}\;p\text{-value}\;(\chi^{2}$)	U(0,1)	1.37 × 10^−10^ ± 4.32 × 10^−10^	[−1.72 × 10^−10^, 4.46 × 10^−10^]
	RC-symmetry p (k = 1)	U(0,1)	0.9999976 ± 0.0000028	[0.9999956, 0.9999996]
	RC-symmetry p (k = 2)	U(0,1)	0.9635 ± 0.0655	[0.9166, 1.0103]
	RC-symmetry p (k = 3)	U(0,1)	0.8470 ± 0.2079	[0.6983, 0.9957]
	H(1)	2	2.0000 ± 0.0000	[2.0000, 2.0000]
	H(2)	4	3.9999 ± 0.0000	[3.9999, 3.9999]
	H(3)	6	5.9997 ± 0.0000	[5.9997, 5.9997]
	H(4)	8	7.9993 ± 0.0001	[7.9992, 7.9994]
	H(5)	10	9.9980 ± 0.0001	[9.9979, 9.9981]
	H(6)	12	11.9923 ± 0.0002	[11.9922, 11.9925]
	Bit p(1)	0.50	0.5000 ± 0.0004	[0.4997, 0.5002]
	zlib ratio	≈0.15	0.1498 ± 0.0000	[0.1498, 0.1499]

Note: Values are reported as the mean ± sample SD and Student-t-based 95% CI calculated over 10 independent 500,000-base DNA streams for each setting. Stream-level tables in Appendix F are rounded for display, whereas the summary statistics in the main text were calculated from full-precision outputs. *p*-values are interpreted as descriptive structural indicators and were not used as standalone pass/fail decisions or as evidence of cryptographic security. Symmetric CI calculations may produce values outside the natural range for metrics that are theoretically non-negative or bounded within [0, 1]; such limits should be interpreted within the physical range of the corresponding metric.

**Table 8:** Summary of exact-match k-mer leakage control for R1, R1-ext, R2, and R3.

Regime	Reference Corpus	Generated Streams	k = 32 hits	k = 48 hits	k = 64 hits	Result
R1	1 M bases	10 × 500 k bases	0	0	0	No leakage detected
R1-ext	1 M bases	10 × 500 k bases	0	0	0	No leakage detected
R2	1 M bases	10 × 500 k bases	0	0	0	No leakage detected
R3	—	—	—	—	—	Not applicable

Note: The exact-match k-mer leakage control was performed for k = 32, 48, and 64 using the corresponding 1 M-base reference/training DNA corpus in each of the R1, R1-ext, and R2 settings. The R3 setting was not included because it does not use an external training or reference corpus. The absence of exact-match hits provides evidence against direct long-fragment copying under the tested criteria. However, it should not be interpreted as formal proof that all possible forms of memorization are absent.

**Table 9:** Bit-domain Hamming and DNA-domain mismatch-based multi-stream independence results calculated over 45 stream pairs under the R1, R1-ext, R2, and R3 settings.

Metric		R1	R1-ext	R2	R3
Bit Domain- pooled Hamming $\left(H_{0}:\;p\;=\;0.5\right)$	Stream pairs	45	45	45	45
	Total n	45,000,000	45,000,000	45,000,000	45,000,000
	Pooled Hamming $\hat{p}$	0.500035	0.499987	0.500078	0.500054
	95% CI	[0.499888, 0.500181]	[0.499841, 0.500133]	[0.499932, 0.500225]	[0.499908, 0.500200]
	*p* vs. 0.5	0.643	0.864	0.292	0.471
DNA Domain- pooled mismatch $\left(H_{0}:\;p\;=\;0.5\right)$	Stream pairs	45	45	45	45
	Total n	22,500,000	22,500,000	22,500,000	22,500,000
	Pooled mismatch $\hat{p}$	0.732184	0.740847	0.749915	0.750116
	95% CI	[0.732001, 0.732367]	[0.740666, 0.741028]	[0.749736, 0.750094]	[0.749937, 0.750295]
	*p* vs. 0.75	<1 × 10^−300^	<1 × 10^−300^	0.350	0.204

**Table 10:** Summary of component sensitivity and failure-oriented stress control under the R3 setting.

Variant	Modified Component	GC Ratio, Mean ± SD	Max. Homopolymer, Mean ± SD	Bit p(1), Mean ± SD	Normalized Rule Entropy, Mean ± SD	Main Observation
fixed-rule	Dynamic DNA-to-bit rule selection was disabled; a single fixed rule was used	0.4984 ± 0.0016	5.0 ± 0.0	0.4905 ± 0.0009	0.0000 ± 0.0000	DNA constraints were preserved; however, bit balance was clearly disrupted and Monobit *p*-values remained very low
no- homopolymer-mask	Homopolymer mask was removed	0.4929 ± 0.0009	18.0 ± 2.0	0.4999 ± 0.0002	0.999998 ± 0.000002	Bit balance was preserved; however, long homopolymer runs emerged
no-lag1-suppression	Lag-1 suppression was disabled	0.4975 ± 0.0009	5.0 ± 0.0	0.5000 ± 0.0001	0.999998 ± 0.000002	No clear degradation was observed in the main GC, homopolymer, or bit-balance metrics
no-constraint	GC balancing, homopolymer mask, and lag-1 suppression were jointly disabled	0.4894 ± 0.0013	20.7 ± 2.1	0.5002 ± 0.0004	0.999998 ± 0.000001	Bit balance was largely preserved, but DNA-level structural compatibility was clearly degraded

**Table 11:** Descriptive summary of empirical performance results for 10 independent streams under CPU-only execution conditions in the R1, R1-ext, R2, and R3 settings.

Regime	Metric	Time (min)	CPU Time (min)	ΔRAM (MB)	Efficiency (Bases/min)
R1	Mean ± SD	28.0719 ± 0.7400	167.8401 ± 4.3422	16.2880 ± 3.6690	17,822.5898 ± 472.5666
	95% CI	[27.5426, 28.6013]	[164.7339, 170.9463]	[13.6634, 18.9126]	[17,484.5360, 18,160.6436]
R1-ext	Mean ± SD	31.3657 ± 0.8979	186.2929 ± 5.0991	23.4950 ± 7.4964	15,952.7817 ± 458.9759
	95% CI	[30.7234, 32.0081]	[182.6452, 189.9406]	[18.1324, 28.8576]	[15,624.4501, 16,281.1132]
R2	Mean ± SD	30.1492 ± 0.2463	180.3740 ± 1.4594	16.9590 ± 3.9924	16,585.1999 ± 135.0661
	95% CI	[29.9730, 30.3254]	[179.3300, 181.4180]	[14.1030, 19.8150]	[16,488.5794, 16,681.8204]
R3	Mean ± SD	26.3361 ± 0.8217	157.1564 ± 4.7000	31.7300 ± 0.9678	19001.6482 ± 581.2117
	95% CI	[25.7483, 26.9239]	[153.7943, 160.5186]	[31.0376, 32.4224]	[18,585.8744, 19,417.4220]

Note: Values are reported as the mean ± sample SD and Student-t-based 95% CI calculated over 10 independent runs for each regime. Time denotes total wall-clock execution time, CPU time denotes cumulative CPU time, and memory denotes the additional memory increase measured relative to the initial state. For R1, R1-ext, and R2, time and resource usage include both training and generation stages. For R3, only generation is measured.

**Table 12:** Comparative summary of selected machine-learning-based PRNG studies.

Feature/Ref.	This Work	[8]	[22]	[25]	[26]	[30]
Model	Dual-head decoder-only Transformer	Decoder-only Transformer	LSTM	DRL + LSTM	GAGAN	UDRL
Output domain	DNA + bitstream	n-bit	Bit sequences	Bit sequences	256-bit output	128-bit blocks
NIST SP 800-22 [16,17]	Yes; 15 tests, 4 settings, 10 × 1 Mbit	Partial; 11/15 tests	Yes	Yes	Yes	Yes
SP 800-90B/[18] ENT [20,21]	Yes; both reported	Not reported	Not reported	Not reported	Partial; GM/T-like standard reported, SP 800-90B/ENT not reported	Partial; entropy-based evaluation reported, SP 800-90B/ENT not reported
DNA- specific metrics	GC ratio, homopolymer limit, 3-mer behavior, RC symmetry, block entropy, Lempel–Ziv-based complexity	Not reported	Not reported	Not reported	Not reported	Not reported
Multi-stream/leakage control	Yes; Hamming, mismatch, k-mer leakage	Partial; prediction attack analysis	Not reported	Not reported	Partial; Hamming/sensitivity analysis	Partial; PNB/leakage analysis reported
Performance analysis	Yes; time, CPU, memory, efficiency, theoretical complexity	Partial; training accuracy and loss curves	Partial; computational complexity reported	Partial; training/reward curves	Yes; inference time and throughput reported	Partial; test and analysis reports
Main distinction	DNA-local generation, in-generation constraint control, dynamic DNA-to-bit mapping, multilayer validation	No DNA-local validation	No DNA-specific analysis	No SP 800-90B, ENT, DNA-specific metrics, or leakage analysis	No multi-stream independence, DNA-specific validation, or regime-disentangled evaluation	No DNA-local generation, DNA-specific metrics, or in-generation constraint management

## Data Availability

The code and reproducibility materials supporting the findings of this study are publicly available in the GitHub repository dna-local-prng-entropy (https://github.com/alevkaya-byte/dna-local-prng-entropy, accessed on 6 April 2026). Publicly available materials include the main generation scripts, recorded run seeds, and the generated DNA outputs, corresponding bitstreams, metadata files, and rule-trace files for the independent streams reported under all three regimes. The public-source genomic input used in the real-data regime is described in the manuscript.

## Abbreviations

The following abbreviations are used in this manuscript:

PRNG	Pseudorandom Number Generator
DNA	Deoxyribonucleic Acid
NIST	National Institute of Standards and Technology
ENT	The Pseudorandom Number Sequence Test Program
GC	Guanine–Cytosine
RC	Reverse Complement
KV	Key–Value
SDPA	Scaled Dot-Product Attention
CSPRNG	Cryptographically Secure Pseudorandom Number Generator
KDF	Key-Derivation Function

## Appendix A

**Table A1:** Extended inventory of representative PRNG and DNA-coding approaches.

Ref.	Family	Method/Approach	Output Space	Training/ Input	Constraint Handling	Dynamic DNA → Bit Mapping	Validation Scope	Reproducibility/Note
**[3]**	DNA coding/ codec	DNA-Aeon (arithmetic coding)	Bit → DNA; dynamic	No training; codec design	Codebook-based	—	DNA constraint compatibility	Not a PRNG; no in-generation constraints
**[4]**	DNA coding/ codec	Explorer (De Bruijn graph)	Bit → DNA; dynamic	No training for Explorer; optional trained Codeformer decoder	Graph-guided / in-generation	—	DNA constraint compatibility	Not a PRNG; no in-generation constraints
**[5]**	DNA coding	Dual-rule rotational coding with chaotic GC control	Bit → DNA; dynamic	No training; chaotic map	Rule-based / chaotic GC control	Partial (2 rules)	Bioinformatics comparison	Not a PRNG; no in-generation constraints
**[6]**	Evaluation/model	DNA storage digital twin	Storage model	—	—	—	DNA error/bias metrics	Does not propose a PRNG/codec
**[8]**	Transformer PRNG	Decoder-only; LCG/MT simulation with CoT	Bit; dynamic	Synthetic PRNG sequences; supervised	—	—	NIST SP 800-22 (most tests); MT prediction	Limited seed/protocol reporting; not DNA-native
**[11]**	Transformer PRNG	Decoder-only; learning LCG regularities	Bit; dynamic	LCG sequences; variety of moduli	—	—	Prediction/generalization experiments	Limited seed/protocol reporting; not DNA-native
**[12]**	DNA coding	Dynamic DNA coding (image)	Bit → DNA; dynamic	Trained/chaotic components	Post	Present	NIST; application metrics	Not a PRNG; no in-generation constraints
**[13]**	DNA coding	Eight-base DNA permutation/diffusion	Bit → DNA; dynamic	Chaotic parameters	Post	Variable	Entropy, etc.; limited	Fixed/narrow rule sets; no unified protocol
**[34]**	DNA key (GA)	DNA key strengthening with GA	DNA; dynamic	External random DNA generator	Post	—	Frequency; gap; fitness	No NIST/90B; dependent on external RNG
**[22]**	DL-PRNG	LSTM + SHA-2	Bit; static	Digits of π + seed	No additional constraints	—	NIST SP 800-22	Seed reproducible; no DNA
**[24]**	DL-PRNG	GAN (Dense/Conv1D)	Bit; dynamic	256-bit seed; framework	—	—	NIST SP 800-22	Seed reproducible; limited security analysis
**[25]**	RL-PRNG	PPO-trained LSTM (POMDP)	Bit; dynamic	Random seed (N = 2/5/10)	—	—	NIST SP 800-22	Open source; reproducible
**[26]**	DL-PRNG	GA-optimized GAN (recursive)	Bit; dynamic	256-bit seed; GA optimization	—	—	NIST; GM/T; Hamming; BCR	Extensive reports; GPU/GA cost
**[27]**	DL-PRNG	Predictive-GAN (seed + counter)	Bit; dynamic	Counter + seed	—	—	NIST SP 800-22 (repeated)	Generation without dataset; no DNA
**[28]**	DL-PRNG	WGAN-GP (end-to-end; ONNX)	Bit; dynamic	Based on MT outputs	—	—	NIST SP 800-22 (multiple repetitions)	Platform-independent; no DNA
**[31]**	DNA + Chaos + CNN	Pre-trained CNN + chaos + DNA coding	Bit → DNA; dynamic	CNN (ImageNet) + images	Post	Variable	NIST; entropy	Data/training-dependent; no in-generation constraints
**[29]**	RL-PRNG	RL agent + LSTM + CNN	Bit; dynamic	Initial vector; no external data	—	—	NIST; correlation	Limited security and hardware reporting
**[30]**	RL-PRNG	UDRL (upside-down RL)	Bit; dynamic	128-bit seed; no data	—	—	NIST; entropy; PNB; Hamming	No dataset required; hardware/time metrics limited
**This work**	Transformer PRNG	Dual-head decoder-only Transformer	DNA → Bit; dynamic	Real DNA/synthetic DNA/no-ref	In-generation	Present	NIST SP 800-22 + SP 800-90B + DNA-specific metrics	Seeded and traceable; DNA-native unified protocol

**Table A2:** Extended architectural comparison between the original Transformer and the proposed DNA-PRNG core.

Component/ Feature	Original Transformer (2017) [7]	Rationale for Use	Proposed PRNG Core	Rationale for Use
**Purpose**	Machine translation (seq2seq translation, enc + dec)	Convert a source sentence into a target language	DNA-native dual-head PRNG with dynamic DNA-to-bit rule mapping	Architecture repurposed for autoregressive DNA generation and rule-guided bitstream derivation
**Architecture**	Encoder + decoder (6 + 6)	Encoder extracts semantics from input; decoder generates output	Decoder-only, 3 blocks	Cross-attention is unnecessary; reduces parameters and latency
**Positional** **encoding**	Fixed sinusoidal (sin/cos)	Sequence order injected externally	RoPE (Q, K)	Compatible with autoregressive generation, sliding window, and KV-cache
**Multi-Head** **Attention (MHA)**	8 heads (base), 16 heads (big)	Multiple attention views	4 heads	Good compute/performance trade-off for a 4-symbol output space
**(** $d_{model}$ **)**	512 (base), 1024 (big)	High capacity for language tasks	128	Lightweight configuration for trained and no-ref regimes
**Feed-Forward Network (FFN)/Activation**	Two-layer MLP, (=2048), ReLU	Increases representational power	Two-layer MLP, GELU	Stable lightweight training and generation
**Number of layers**	6 encoder + 6 decoder	Deep structure for complex sequence patterns	3 blocks	Sufficient under the reported regimes while keeping the core lightweight
**Normalization/Residual**	LayerNorm + residual connections	Stabilizes learning	Pre-LN + residual	Matches implementation and supports stable forward passes
**Dropout**	0.1–0.3	Prevents overfitting	0.1 in the trained model; 0.0 in the no-ref model; inactive at inference	Consistent with trained/no-ref code paths
**Decoder masking**	Mandatory (future mask)	Enables autoregressive generation	Causal SDPA (is_causal = True)	Prevents access to future positions
**KV-cache/Window**	—	—	KV-cache + sliding window ($block_{size}$ = 128)	Reduces latency and memory cost during long generation
**Input representation**	Words (token IDs)	Vectorized via embeddings	DNA token IDs (A/C/G/T → 0–3)	Supports DNA-local autoregressive modeling
**Training data (regimes)**	Large parallel corpus	Parameter optimization for translation	R1: real DNA; R2: synthetic DNA; R3: no-ref	R1/R2 trained; R3 generated without training data
**Optimization (R1/R2)**	Adam/AdamW	Widely adopted optimizers	AdamW, lr = 3 × 10^−4^, wd = 0.01, batch = 256, epoch = 1	Lightweight training configuration
**Output heads**	Single vocabulary head	Predicts next token	Dual heads: base-head (4), rule-head (8)	Joint next-base prediction and dynamic rule distribution modeling
**Channel constraints**	—	Not required for language tasks	GC ≈ 0.5, homopolymer ≤ 5, lag-1 damping; trained code additionally uses bigram/trimer smoothing	Supports DNA-compatible generation behavior

## Appendix B

**Table A3:** Flow-level core performance summary for R1, R1-ext, R2 and R3 excluding I/O and downstream analysis.

Regime	Flow ID	Run_Seed	Time (min)	CPU Time (min)	ΔRAM (MB)	Efficiency (Bases/min)	Most-Used Rule
R1	Run1	3777576837658288813	27.578	164.919	17.03	18,130.1	R1
	Run2	2996532206919239426	27.013	161.594	14.23	18,509.7	R2
	Run3	9015237046617222238	28.513	170.422	20.13	17,535.9	R4
	Run4	6751083079595914137	29.050	173.598	13.00	17,211.6	R7
	Run5	6605099016974130776	28.595	170.797	12.56	17,485.8	R3
	Run6	2901449802581831812	28.605	171.092	13.26	17,479.2	R4
	Run7	8148888929366623013	27.330	163.516	21.99	18,295.1	R8
	Run8	4343358777370873001	28.786	171.955	14.64	17,369.8	R3
	Run9	2575375486695530171	28.069	167.900	14.38	17,813.1	R8
	Run10	8122644735018647104	27.181	162.608	21.66	18,395.4	R7
R1-ext	Run1	7625999889054559659	30.733	182.599	24.96	16,268.9	R5
	Run2	314573498243332620	31.283	185.664	31.94	15,983.1	R2
	Run3	764733146881143348	31.283	185.944	13.99	15,983.3	R1
	Run4	251898976151475512	31.613	187.132	13.72	15,816.4	R4
	Run5	7498231141980839004	29.772	177.353	14.58	16,794.1	R6
	Run6	3738891002146033876	31.005	184.407	27.80	16,126.6	R6
	Run7	2941200863645545546	30.617	182.153	32.41	16,330.7	R6
	Run8	2607212318199907590	32.442	192.742	19.19	15,412.0	R7
	Run9	2875944969232760973	32.347	191.882	26.38	15,457.4	R5
	Run10	6067218175503377371	32.562	193.053	29.98	15,355.3	R0
R2	Run1	2659341318112875182	30.615	183.130	20.84	16,332.0	R6
	Run2	3246168301735005977	30.436	182.105	13.91	16,428.1	R3
	Run3	6391463415693057775	30.294	181.220	13.62	16,504.9	R6
	Run4	542021218614881257	30.115	180.143	19.27	16,603.2	R2
	Run5	3365475962873395619	30.046	179.765	21.91	16,641.1	R3
	Run6	1949493743774670690	30.129	180.220	20.23	16,595.3	R1
	Run7	3238582911584386627	30.118	180.184	20.73	16,601.2	R4
	Run8	3765494611416181708	30.075	179.955	13.45	16,624.9	R8
	Run9	1827248064715351053	29.776	178.156	11.01	16,792.2	R6
	Run10	7156019210394983219	29.888	178.862	14.62	16,729.1	R1
R3	Run1	8687970821042739881	25.888	154.560	31.65	19,313.6	R7
	Run2	2717860915976751978	27.841	165.042	33.38	17,959.2	R5
	Run3	4168663315303670174	25.641	152.989	31.06	19,499.7	R2
	Run4	1899467523397611477	26.027	155.134	32.54	19,211.1	R5
	Run5	669719007328324969	26.290	156.853	31.68	19,018.8	R7
	Run6	6078085842953122236	25.601	152.979	30.16	19,530.2	R5
	Run7	8506520864525150646	25.401	151.838	31.42	19,684.5	R7
	Run8	7744048495857627520	26.937	160.909	30.75	18,561.9	R2
	Run9	28728216386926652	27.455	164.121	31.91	18,211.4	R6
	Run10	2343752742164881949	26.280	157.138	32.75	19,026.0	R5

## Appendix C

**Table A4:** R1, R1-EXT, R2 and R3 NIST SP 800-22 *p*-values for 10 independent runs.

Regime	Test/Flow	Run1	Run2	Run3	Run4	Run5	Run6	Run7	Run8	Run9	Run10
R1	T1 Monobit	0.574116	0.947378	0.972877	0.171939	0.941010	0.980853	0.114107	0.491453	0.269593	0.413357
	T2 Block Frequency *	0.669543	0.177556	0.976522	0.819986	0.915793	0.089008	0.345831	0.855791	0.220493	0.532428
	T3 Runs	0.982196	0.265277	0.279252	0.801144	0.976063	0.587818	0.830160	0.621640	0.894020	0.224240
	T4 Longest Run	0.412683	0.717564	0.991486	0.997963	0.847873	0.216029	0.060961	0.573909	0.133437	0.672628
	T5 Rank	0.272268	0.529120	0.898560	0.152231	0.441833	0.365876	0.656461	0.487610	0.851033	0.347866
	T6 DFT	0.127679	0.502925	0.393423	0.613759	0.832839	0.762020	0.192547	0.626711	0.192547	0.056296
	T7 Non-overlapping Template *	0.095713	0.691427	0.990490	0.283391	0.449521	0.882317	0.265165	0.246289	0.433015	0.491737
	T8 Overlapping Template	0.760016	0.391747	0.455295	0.902004	0.839479	0.094000	0.780932	0.301322	0.925462	0.580660
	T9 Universal	0.294271	0.726637	0.503237	0.098713	0.047189	0.773077	0.243926	0.439691	0.308580	0.924031
	T10 Linear Complexity	0.434030	0.120095	0.518555	0.206627	0.634534	0.149757	0.320940	0.768136	0.473276	0.958357
	T11 Serial *	0.068368	0.802227	0.032145	0.265820	0.907015	0.546542	0.511602	0.689968	0.490083	0.344284
	T12 Approximate Entropy	0.348051	0.478736	0.875891	0.908812	0.752068	0.015260	0.950942	0.690076	0.686052	0.602374
	T13 Cumulative Sums (Backward)	0.964088	0.534794	0.911652	0.307699	0.876640	0.776553	0.134201	0.561361	0.325975	0.191824
	T14 Random Excursions *	0.940605	0.087099	0.836160	0.327066	0.564418	0.358256	0.442430	0.814501	0.263074	0.460295
	T15 Random Excursions Variant *	0.618112	0.648884	0.849951	0.840036	0.502843	0.073160	0.872932	0.553694	0.796987	0.967530
R1-EXT	T1 Monobit	0.876033	0.299270	0.023451	0.918757	0.465390	0.227817	0.176375	0.677410	0.192916	0.893403
	T2 Block Frequency *	0.688866	0.518367	0.315215	0.405310	0.273553	0.793153	0.435942	0.350540	0.402832	0.421235
	T3 Runs	0.172575	0.914727	0.730194	0.399794	0.018546	0.185321	0.871170	0.192293	0.085377	0.964890
	T4 Longest Run	0.701222	0.904868	0.990511	0.112640	0.840767	0.773268	0.466527	0.535885	0.881214	0.842003
	T5 Rank	0.400857	0.934882	0.122663	0.813768	0.911907	0.248056	0.988609	0.507021	0.976758	0.498481
	T6 DFT	0.022314	0.686381	0.312769	0.769024	0.011924	0.783087	0.468480	0.948782	0.948782	0.479815
	T7 Non-overlapping Template *	0.483935	0.081419	0.987610	0.472613	0.273594	0.949683	0.419182	0.143944	0.682696	0.584531
	T8 Overlapping Template	0.904326	0.278928	0.254728	0.474896	0.487261	0.538331	0.684371	0.473233	0.904803	0.593225
	T9 Universal	0.263026	0.064627	0.833899	0.200944	0.737001	0.126080	0.976729	0.205922	0.544305	0.770810
	T10 Linear Complexity	0.390649	0.932794	0.911430	0.093254	0.435055	0.054720	0.771536	0.640324	0.975377	0.641268
	T11 Serial *	0.010475	0.244475	0.083704	0.683724	0.235186	0.794645	0.881126	0.951755	0.349147	0.484959
	T12 Approximate Entropy	0.902937	0.872248	0.572556	0.696465	0.985675	0.341278	0.776160	0.271060	0.643875	0.908190
	T13 Cumulative Sums (Backward)	0.648534	0.029456	0.036550	0.728023	0.469716	0.227752	0.214357	0.771929	0.196254	0.913034
	T14 Random Excursions *	0.525969	0.065370	0.012732	0.892020	0.334134	0.934651	0.051210	0.021177	0.723167	0.302438
	T15 Random Excursions Variant *	0.272304	0.929763	0.227610	0.461714	0.417111	0.244214	0.514598	0.071589	0.339418	0.822266
R2	T1 Monobit	0.846176	0.998404	0.022608	0.344149	0.334044	0.047256	0.605854	0.807231	0.370253	0.221708
	T2 Block Frequency *	0.961222	0.734024	0.761550	0.328885	0.653973	0.887630	0.694811	0.932147	0.543368	0.894749
	T3 Runs	0.145936	0.253454	0.828053	0.456335	0.542568	0.420278	0.360857	0.984091	0.801662	0.590926
	T4 Longest Run	0.454939	0.027110	0.851361	0.621484	0.574523	0.094218	0.966306	0.146381	0.214736	0.580577
	T5 Rank	0.971163	0.473230	0.609144	0.312749	0.300130	0.135480	0.862600	0.435200	0.882449	0.480366
	T6 DFT	0.057491	0.912315	0.373394	0.070638	0.116601	0.948782	0.934178	0.378341	0.388356	0.013919
	T7 Non-overlapping Template *	0.533261	0.530300	0.648813	0.592512	0.826356	0.647508	0.299097	0.318556	0.944411	0.342988
	T8 Overlapping Template	0.054371	0.792526	0.061094	0.845211	0.721246	0.985753	0.683012	0.238079	0.017992	0.229354
	T9 Universal	0.305657	0.276097	0.040213	0.923409	0.336758	0.198378	0.532490	0.519868	0.407002	0.176780
	T10 Linear Complexity	0.618159	0.188254	0.160839	0.604675	0.233668	0.510675	0.896151	0.532704	0.449326	0.183131
	T11 Serial *	0.059399	0.675536	0.664156	0.626823	0.724078	0.967183	0.144986	0.097246	0.866850	0.087061
	T12 Approximate Entropy	0.112163	0.230017	0.420250	0.302175	0.892372	0.424580	0.518228	0.377351	0.131236	0.297065
	T13 Cumulative Sums (Backward)	0.987844	0.866193	0.023874	0.634720	0.429552	0.080534	0.458105	0.934381	0.545849	0.428818
	T14 Random Excursions *	0.631132	0.937194	0.868588	0.515450	0.328942	0.784265	0.854095	0.426371	0.077111	0.199304
	T15 Random Excursions Variant *	0.206347	0.486963	0.146903	0.287350	0.916010	0.144244	0.159694	0.773638	0.106827	0.991227
R3	T1 Monobit	0.926698	0.437749	0.874457	0.540538	0.191553	0.832107	0.524776	0.231697	0.858723	0.578211
	T2 Block Frequency *	0.861260	0.904103	0.627879	0.990253	0.314213	0.391705	0.111155	0.950025	0.474425	0.360097
	T3 Runs	0.860301	0.869257	0.917189	0.990724	0.994978	0.780978	0.068821	0.456011	0.267013	0.689384
	T4 Longest Run	0.604606	0.632427	0.034811	0.051520	0.222293	0.787522	0.137809	0.871183	0.563663	0.520913
	T5 Rank	0.233986	0.202430	0.772287	0.262833	0.768670	0.919391	0.772087	0.877492	0.495204	0.919391
	T6 DFT	0.832839	0.978037	0.727306	0.408863	0.790145	0.790145	0.363621	0.588217	0.532620	0.215403
	T7 Non-overlapping Template *	0.206548	0.990923	0.818175	0.929475	0.811983	0.503267	0.728726	0.649169	0.479238	0.010210
	T8 Overlapping Template	0.598509	0.999010	0.437266	0.236232	0.507910	0.155461	0.763075	0.842145	0.068121	0.026088
	T9 Universal	0.918352	0.058300	0.651230	0.395361	0.871172	0.589699	0.904884	0.335321	0.194210	0.624306
	T10 Linear Complexity	0.768922	0.560721	0.017217	0.540099	0.456682	0.846863	0.430851	0.612810	0.966705	0.462924
	T11 Serial *	0.484166	0.344815	0.432245	0.589628	0.876529	0.721337	0.609739	0.701161	0.337268	0.345081
	T12 Approximate Entropy	0.408452	0.795013	0.527341	0.657171	0.908966	0.972334	0.121313	0.670852	0.566861	0.708757
	T13 Cumulative Sums (Backward)	0.943118	0.270859	0.986478	0.891349	0.213052	0.504049	0.715808	0.350103	0.771929	0.373520
	T14 Random Excursions *	0.469141	0.835147	0.037276	0.142782	0.610450	0.490443	0.929087	0.271992	0.939207	0.741596
	T15 Random Excursions Variant *	0.743347	0.383574	0.084301	0.933395	0.257587	0.657491	0.792408	0.853559	0.936042	0.266002

* For tests with multiple parameter-dependent or multiple *p*-value outputs, the reported value corresponds to the predefined compact-summary rule: M = 1000 for Block Frequency, template 000000001 for Non-overlapping Template, the second *p*-value for Serial, the backward direction for Cumulative Sums, the +4 state for Random Excursions, and the +9 state for Random Excursions Variant.

## Appendix D

**Table A5:** R1, R1-EXT, R2 and R3 SP 800-90B-inspired entropy, IID, and health-test results.

Regime	Test		Run1	Run2	Run3	Run4	Run5	Run6	Run7	Run8	Run9	Run10
R1	Entropy tests	p(1)	0.500281	0.500033	0.499983	0.500683	0.500037	0.499988	0.500790	0.499656	0.499448	0.500409
		H_MCV (bit/bit)	0.999189	0.999905	0.999951	0.998031	0.999893	0.999965	0.997722	0.999008	0.998408	0.998820
		H_min (t-tuple, bit/bit)	0.997047	0.996637	0.996121	0.996849	0.995758	0.996364	0.996698	0.995728	0.996557	0.996470
		H_Markov(1) (bit/bit)	0.999188	0.998395	0.998441	0.998029	0.999892	0.999220	0.997721	0.999007	0.998407	0.998246
		H_Collision H2 (bit/bit)	1.000000	1.000000	1.000000	0.999997	1.000000	1.000000	0.999996	0.999999	0.999998	0.999999
		H_Compression LB (zlib, bit/bit)	1.000000	1.000000	1.000000	1.000000	1.000000	1.000000	1.000000	1.000000	1.000000	1.000000
	IID and health tests	Monobit p	0.574116	0.947378	0.972877	0.171939	0.941010	0.980853	0.114107	0.491453	0.269593	0.413357
		Runs p	0.982196	0.265277	0.279252	0.801144	0.976063	0.587818	0.830160	0.621640	0.894020	0.224240
		$I(X_{t};\;X_{t-1})$ [bits]	0.000000	0.000001	0.000001	0.000000	0.000000	0.000000	0.000000	0.000000	0.000000	0.000001
		RCT max run (threshold ≤ 40)	20 (PASS)	21 (PASS)	19 (PASS)	19 (PASS)	20 (PASS)	18 (PASS)	27 (PASS)	22 (PASS)	21 (PASS)	20 (PASS)
		APT worst window (index, k/512, *p*-value)	#1137; k = 291/512; *p* = 0.00226; PASS	#1338; k = 293/512; *p* = 0.00123; PASS	#1598; k = 298/512; *p* = 0.000237; PASS	#683; k = 217/512; *p* = 0.000652; PASS	#1115; k = 297/512; *p* = 0.000335; PASS	#446; k = 301/512; *p* = 8.06 × 10^−5^; PASS	#35; k = 208/512; *p* = 2.55 × 10^−5^; PASS	#170; k = 292/512; *p* = 0.00168; PASS	#616; k = 219/512; *p* = 0.00123; PASS	#1049; k = 215/512; *p* = 0.000335; PASS
R1-ext	Entropy tests	p(1)	0.500078	0.500519	0.501133	0.499949	0.500365	0.500603	0.500676	0.500208	0.500651	0.500067
		H_MCV (bit/bit)	0.999775	0.998503	0.996735	0.999853	0.998947	0.998261	0.998051	0.999400	0.998123	0.999807
		H_min (t-tuple, bit/bit)	0.994281	0.995290	0.995713	0.995275	0.994131	0.996813	0.996682	0.995230	0.995200	0.996849
		H_Markov(1) (bit/bit)	0.998032	0.998502	0.996733	0.998784	0.996610	0.998088	0.998049	0.998119	0.997518	0.999805
		H_Collision H2 (bit/bit)	1.000000	0.999998	0.999993	1.000000	0.999999	0.999998	0.999997	1.000000	0.999998	1.000000
		H_Compression LB (zlib, bit/bit)	1.000000	1.000000	1.000000	1.000000	1.000000	1.000000	1.000000	1.000000	1.000000	1.000000
	IID and health tests	Monobit p	0.876033	0.299270	0.023451	0.918757	0.465390	0.227817	0.176375	0.677410	0.192916	0.893403
		Runs p	0.172575	0.914727	0.730194	0.399794	0.018546	0.185321	0.871170	0.192293	0.085377	0.964890
		$I(X_{t};\;X_{t-1})$ [bits]	0.000001	0.000000	0.000000	0.000001	0.000004	0.000001	0.000000	0.000001	0.000002	0.000000
		RCT max run (threshold ≤ 40)	21 (PASS)	18 (PASS)	20 (PASS)	23 (PASS)	19 (PASS)	20 (PASS)	17 (PASS)	19 (PASS)	21 (PASS)	19 (PASS)
		APT worst window (index, k/512, *p*-value)	#656; k = 291/512; *p* = 0.00226; PASS	#1346; k = 296/512; *p* = 0.000469; PASS	#348; k = 301/512; *p* = 8.06× 10^−5^; PASS	#1776; k = 215/512; *p* = 0.000335; PASS	#1859; k = 215/512; *p* = 0.000335; PASS	#711; k = 308/512; *p* = 4.95 × 10^−6^; PASS	#461; k = 218/512; *p* = 0.0009; PASS	#1843; k = 209/512; *p* = 3.78 × 10^−5^; PASS	#1073; k = 303/512; *p* = 3.78 × 10^−5^; PASS	#554; k = 215/512; *p* = 0.000335; PASS
R2	Entropy tests	p(1)	0.499903	0.500001	0.501140	0.499527	0.500483	0.500992	0.500258	0.500122	0.500448	0.500611
		H_MCV (bit/bit)	0.999720	0.999997	0.996714	0.998636	0.998607	0.997141	0.999256	0.999648	0.998708	0.998238
		H_min (t-tuple, bit/bit)	0.994522	0.996121	0.995683	0.996682	0.997686	0.995970	0.994928	0.995154	0.994958	0.996000
		H_Markov(1) (bit/bit)	0.997905	0.998355	0.996713	0.998638	0.998609	0.997142	0.998681	0.999647	0.998707	0.998237
		H_Collision H2 (bit/bit)	1.000000	1.000000	0.999993	0.999999	0.999999	0.999994	1.000000	1.000000	0.999999	0.999998
		H_Compression LB (zlib, bit/bit)	1.000000	1.000000	1.000000	1.000000	1.000000	1.000000	1.000000	1.000000	1.000000	1.000000
	IID and health tests	Monobit p	0.846176	0.998404	0.022608	0.344149	0.334044	0.047256	0.605854	0.807231	0.370253	0.221708
		Runs p	0.145936	0.253454	0.828053	0.456335	0.542568	0.420278	0.360857	0.984091	0.801662	0.590926
		$I(X_{t};\;X_{t-1})$ [bits]	0.000002	0.000001	0.000000	0.000000	0.000000	0.000000	0.000001	0.000000	0.000000	0.000000
		RCT max run (threshold ≤ 40)	22 (PASS)	21 (PASS)	19 (PASS)	19 (PASS)	20 (PASS)	20 (PASS)	19 (PASS)	20 (PASS)	21 (PASS)	23 (PASS)
		APT worst window (index, k/512, *p*-value)	#497; k = 215/512; *p* = 0.000335; PASS	#1210; k = 212/512; *p* = 0.000116; PASS	#625; k = 220/512; *p* = 0.00168; PASS	#532; k = 295/512; *p* = 0.000652; PASS	#1421; k = 292/512; *p* = 0.00168; PASS	#1304; k = 215/512; *p* = 0.000335; PASS	#517; k = 306/512; *p* = 1.14 × 10^−5^; PASS	#970; k = 218/512; *p* = 0.0009; PASS	#1818; k = 296/512; *p* = 0.000469; PASS	#1660; k = 302/512; *p* = 5.54 × 10^−5^; PASS
R3	Entropy tests	p(1)	0.500046	0.500388	0.500079	0.500306	0.499347	0.500106	0.499682	0.499402	0.499911	0.500278
		H_MCV (bit/bit)	0.999867	0.998881	0.999772	0.999117	0.998117	0.999694	0.999083	0.998276	0.999743	0.999198
		H_min (t-tuple, bit/bit)	0.996167	0.996819	0.996167	0.996394	0.994732	0.997275	0.994928	0.994853	0.994071	0.995350
		H_Markov(1) (bit/bit)	0.999745	0.998879	0.999774	0.999119	0.998116	0.999600	0.997376	0.998275	0.998398	0.999197
		H_Collision H2 (bit/bit)	1.000000	0.999999	1.000000	0.999999	0.999998	1.000000	0.999999	0.999998	1.000000	1.000000
		H_Compression LB (zlib, bit/bit)	1.000000	1.000000	1.000000	1.000000	1.000000	1.000000	1.000000	1.000000	1.000000	1.000000
	IID and health tests	Monobit p	0.926698	0.437749	0.874457	0.540538	0.191553	0.832107	0.524776	0.231697	0.858723	0.578211
		Runs p	0.860301	0.869257	0.917189	0.990724	0.994978	0.780978	0.0688205	0.456011	0.267013	0.689384
		$I(X_{t};\;X_{t-1})$ [bits]	0.000000	0.000000	0.000000	0.000000	0.000000	0.000000	0.000002	0.000000	0.000001	0.000000
		RCT max run (threshold ≤ 40)	27 (PASS)	20 (PASS)	21 (PASS)	20 (PASS)	18 (PASS)	20 (PASS)	18 (PASS)	18 (PASS)	20 (PASS)	23 (PASS)
		APT worst window (index, k/512, *p*-value)	#955; k = 295/512; *p* = 0.000652; PASS	#1579; k = 299/512; *p* = 0.000167; PASS	#218; k = 217/512; *p* = 0.000652; PASS	#1431; k = 294/512; *p* = 0.0009; PASS	#1186; k = 299/512; *p* = 0.000167; PASS	#1145; k = 214/512; *p* = 0.000237; PASS	#132; k = 199/512; *p* = 5.34 × 10^−7^; PASS	#1567; k = 292/512; *p* = 0.00168; PASS	#944; k = 295/512; *p* = 0.000652; PASS	#1437; k = 297/512; *p* = 0.000335; PASS

Note: All RCT and APT checks remained within the acceptance range.

## Appendix E

**Table A6:** R1, R1-ext, R2 and R3 ENT test statistics.

Regime	Metric	Run1	Run2	Run3	Run4	Run5	Run6	Run7	Run8	Run9	Run10
R1	Entropy (bits per byte)	7.998503	7.998453	7.998549	7.998579	7.998272	7.998630	7.998564	7.998726	7.998694	7.998462
	Chi-square statistic	259.457	268.001	250.949	245.637	298.516	238.362	248.352	221.413	227.188	265.781
	Arithmetic mean (ideal: 127.5)	127.7228	127.8182	127.3822	127.8139	127.3011	127.4006	127.6763	127.3662	127.3714	127.7491
	Monte Carlo π estimate	3.14227	3.12320	3.14419	3.14381	3.15610	3.14816	3.13843	3.14970	3.14330	3.13997
	Serial correlation coefficient	0.003329	0.000891	0.002626	−0.000355	−0.000289	0.003326	0.001138	0.002030	−0.003179	0.005526
R1-ext	Entropy (bits per byte)	7.998590	7.998598	7.998371	7.998577	7.998582	7.998358	7.998401	7.998457	7.998636	7.998595
	Chi-square statistic	244.420	243.204	280.629	246.136	244.629	284.418	277.278	267.788	235.991	243.245
	Arithmetic mean (ideal: 127.5)	127.2646	127.8210	127.8679	127.2059	127.5383	127.8565	127.8331	127.9599	127.7023	127.3961
	Monte Carlo π estimate	3.14406	3.12563	3.13702	3.15213	3.13011	3.12346	3.13306	3.13050	3.14624	3.14650
	Serial correlation coefficient	0.002862	−0.000997	−0.001591	−0.001028	0.002211	0.004837	−0.001910	0.000066	0.004142	0.001290
R2	Entropy (bits per byte)	7.998508	7.998720	7.998471	7.998629	7.998678	7.998570	7.998533	7.998171	7.998434	7.998508
	Chi-square statistic	257.548	221.982	264.466	237.760	229.502	247.664	254.283	317.313	270.696	258.367
	Arithmetic mean (ideal: 127.5)	127.4776	127.4215	127.7040	127.7753	127.9032	127.5479	127.6526	127.4350	127.3099	127.7128
	Monte Carlo π estimate	3.14176	3.14266	3.15341	3.13459	3.13485	3.14586	3.12960	3.14304	3.13587	3.13600
	Serial correlation coefficient	0.000318	−0.003843	0.001363	−0.002466	0.004851	0.002446	−0.001540	−0.000417	−0.004635	−0.000639
R3	Entropy (bits per byte)	7.998608	7.998592	7.998623	7.998550	7.998590	7.998617	7.998714	7.998375	7.998305	7.998483
	Chi-square statistic	241.025	243.761	239.222	250.929	244.240	239.820	222.429	281.698	293.556	263.016
	Arithmetic mean (ideal: 127.5)	127.2902	127.4808	127.4596	127.7191	127.6245	127.7010	127.3118	127.3744	127.4543	127.5737
	Monte Carlo π estimate	3.14240	3.13203	3.14445	3.12614	3.14445	3.14189	3.14970	3.15520	3.12435	3.13459
	Serial correlation coefficient	−0.002813	0.002570	−0.001346	0.003443	−0.005973	0.002933	−0.000471	0.000145	−0.001226	−0.002661

## Appendix F

**Table A7:** DNA-specific metrics for scenario R1, R1-ext, R2 and R3 (10 independent 500k-base DNA streams).

Regime	Metric	Run1	Run2	Run3	Run4	Run5	Run6	Run7	Run8	Run9	Run10
R1	GC ratio	0.5000	0.4994	0.4999	0.4995	0.4999	0.4999	0.4999	0.4996	0.5000	0.4997
	Homopolymer max	5	5	5	5	5	5	5	5	5	5
	Normalized LZ complexity	0.9652	0.9659	0.9632	0.9684	0.9735	0.9676	0.9695	0.9553	0.9747	0.9611
	3-mer *p*-value ($\chi^{2}$)	0.0	0.0	0.0	0.0	0.0	0.0	0.0	0.0	0.0	0.0
	RC-symmetry p ((k = 1))	0.9996	0.6335	0.9964	0.6037	0.9871	0.9887	0.9857	0.8395	0.9966	0.8526
	RC-symmetry p ((k = 2))	3.91 × 10^−82^	3.83 × 10^−74^	5.19 × 10^−16^	1.40 × 10^−122^	1.07 × 10^−118^	1.26 × 10^−35^	1.20 × 10^−3^	1.28 × 10^−141^	1.31 × 10^−85^	4.54 × 10^−35^
	RC-symmetry p ((k = 3))	2.48 × 10^−165^	9.21 × 10^−227^	3.96 × 10^−33^	1.39 × 10^−295^	7.15 × 10^−245^	4.18 × 10^−100^	1.80 × 10^−17^	0.0	1.21 × 10^−213^	6.01 × 10^−114^
	(H(1))	2.0000	2.0000	2.0000	2.0000	2.0000	2.0000	2.0000	2.0000	2.0000	2.0000
	(H(2))	3.9851	3.9860	3.9855	3.9892	3.9873	3.9845	3.9874	3.9814	3.9876	3.9842
	(H(3))	5.9643	5.9667	5.9631	5.9738	5.9721	5.9641	5.9701	5.9513	5.9732	5.9599
	(H(4))	7.9361	7.9416	7.9316	7.9529	7.9543	7.9379	7.9473	7.9088	7.9563	7.9267
	(H(5))	9.8997	9.9098	9.8909	9.9254	9.9321	9.9049	9.9181	9.8547	9.9358	9.8841
	(H(6))	11.8492	11.8643	11.8353	11.8839	11.9007	11.8595	11.8775	11.7824	11.9067	11.8265
	Bit p(1)	0.5003	0.5000	0.5000	0.5007	0.5000	0.5000	0.5008	0.4997	0.4994	0.5004
	zlib ratio	0.1499	0.1499	0.1497	0.1497	0.1498	0.1498	0.1500	0.1499	0.1499	0.1498
R1-ext	GC ratio	0.4995	0.4996	0.4995	0.4989	0.4995	0.4993	0.4989	0.4996	0.4991	0.4994
	Homopolymer max	5	5	5	5	5	5	5	5	5	5
	Normalized LZ complexity	0.9744	0.9735	0.9723	0.9681	0.9760	0.9733	0.9700	0.9752	0.9706	0.9751
	3-mer *p*-value ($\chi^{2}$)	0.0	0.0	0.0	0.0	0.0	0.0	0.0	0.0	0.0	0.0
	RC-symmetry p ((k = 1))	0.5445	0.6921	0.5686	0.0778	0.6504	0.2960	0.0676	0.5903	0.1411	0.3629
	RC-symmetry p ((k = 2))	1.77 × 10^−32^	1.89 × 10^−84^	5.60 × 10^−52^	2.56 × 10^−102^	2.35 × 10^−48^	2.54 × 10^−8^	4.81 × 10^−55^	1.73 × 10^−81^	1.61 × 10^−104^	1.60 × 10^−3^
	RC-symmetry p ((k = 3))	5.60 × 10^−86^	4.04 × 10^−254^	8.60 × 10^−128^	2.20 × 10^−290^	1.66 × 10^−157^	3.59 × 10^−68^	5.24 × 10^−214^	1.27 × 10^−188^	3.23 × 10^−281^	4.29 × 10^−50^
	(H(1))	2.0000	2.0000	2.0000	2.0000	2.0000	2.0000	2.0000	2.0000	2.0000	2.0000
	(H(2))	3.9869	3.9863	3.9836	3.9838	3.9899	3.9862	3.9866	3.9858	3.9879	3.9876
	(H(3))	5.9728	5.9710	5.9658	5.9657	5.9787	5.9713	5.9713	5.9710	5.9740	5.9745
	(H(4))	7.9570	7.9534	7.9460	7.9452	7.9658	7.9547	7.9538	7.9551	7.9579	7.9602
	(H(5))	9.9387	9.9326	9.9231	9.9203	9.9505	9.9352	9.9326	9.9371	9.9380	9.9435
	(H(6))	11.9117	11.9028	11.8912	11.8828	11.9268	11.9064	11.8992	11.9114	11.9070	11.9178
	Bit p(1)	0.5001	0.5005	0.5011	0.4999	0.5004	0.5006	0.5007	0.5002	0.5007	0.5001
	zlib ratio	0.1499	0.1499	0.1498	0.1498	0.1499	0.1498	0.1499	0.1498	0.1497	0.1499
R2	GC ratio	0.5001	0.5000	0.5000	0.4999	0.5000	0.5000	0.4999	0.5000	0.5000	0.5000
	Homopolymer max	5	5	5	5	5	5	5	5	5	5
	Normalized LZ complexity	0.9855	0.9857	0.9865	0.9858	0.9870	0.9862	0.9863	0.9868	0.9866	0.9865
	3-mer *p*-value ($\chi^{2}$)	8.20 × 10^−100^	1.65 × 10^−29^	5.23 × 10^−19^	1.60 × 10^−7^	2.33 × 10^−28^	3.66 × 10^−116^	5.67 × 10^−32^	1.83 × 10^−37^	2.14 × 10^−66^	1.46 × 10^−48^
	RC-symmetry p ((k = 1))	0.9831	1.0000	0.9980	0.9916	1.0000	0.9947	0.9872	1.0000	0.9999	0.9972
	RC-symmetry p ((k = 2))	8.19 × 10^−12^	0.0138	0.0018	2.28 × 10^−4^	1.06 × 10^−10^	3.20 × 10^−19^	0.4087	9.90 × 10^−5^	1.19 × 10^−25^	1.30 × 10^−6^
	RC-symmetry p ((k = 3))	5.19 × 10^−20^	0.0085	6.90 × 10^−4^	1.73 × 10^−6^	1.24 × 10^−16^	6.93 × 10^−33^	0.1467	1.62 × 10^−6^	3.23 × 10^−45^	1.92 × 10^−9^
	(H(1))	2.0000	2.0000	2.0000	2.0000	2.0000	2.0000	2.0000	2.0000	2.0000	2.0000
	(H(2))	3.9995	3.9998	3.9999	3.9999	3.9998	3.9995	3.9998	3.9998	3.9997	3.9997
	(H(3))	5.9990	5.9996	5.9997	5.9998	5.9996	5.9989	5.9996	5.9995	5.9993	5.9994
	(H(4))	7.9984	7.9991	7.9993	7.9994	7.9991	7.9981	7.9991	7.9991	7.9987	7.9989
	(H(5))	9.9968	9.9977	9.9979	9.9981	9.9977	9.9964	9.9977	9.9976	9.9972	9.9974
	(H(6))	11.9907	11.9918	11.9920	11.9921	11.9917	11.9901	11.9919	11.9919	11.9915	11.9913
	Bit p(1)	0.4999	0.5000	0.5011	0.4995	0.5005	0.5010	0.5003	0.5001	0.5004	0.5006
	zlib ratio	0.1499	0.1498	0.1499	0.1499	0.1499	0.1498	0.1498	0.1498	0.1498	0.1498
R3	GC ratio	0.5000	0.5000	0.5000	0.5000	0.5000	0.5000	0.5000	0.5000	0.5000	0.5000
	Homopolymer max	5	5	5	5	5	5	5	5	5	5
	Normalized LZ complexity	0.9859	0.9867	0.9871	0.9866	0.9865	0.9861	0.9864	0.9864	0.9857	0.9865
	3-mer *p*-value ($\chi^{2}$)	1.37 × 10^−9^	3.96 × 10^−18^	2.29 × 10^−13^	2.71 × 10^−15^	5.53 × 10^−16^	1.70 × 10^−15^	8.41 × 10^−15^	6.38 × 10^−25^	5.48 × 10^−14^	1.20 × 10^−23^
	RC-symmetry p ((k = 1))	1.0000	1.0000	1.0000	1.0000	1.0000	1.0000	1.0000	1.0000	1.0000	1.0000
	RC-symmetry p ((k = 2))	0.9856	0.8860	0.9994	0.9986	0.8075	0.9998	0.9998	0.9999	0.9994	0.9586
	RC-symmetry p ((k = 3))	0.9997	0.9812	0.9256	0.9926	0.9542	0.9857	0.4414	0.6529	0.9616	0.5748
	(H(1))	2.0000	2.0000	2.0000	2.0000	2.0000	2.0000	2.0000	2.0000	2.0000	2.0000
	(H(2))	3.9999	3.9999	3.9999	3.9999	3.9999	3.9999	3.9999	3.9999	3.9999	3.9999
	(H(3))	5.9998	5.9997	5.9997	5.9997	5.9997	5.9997	5.9997	5.9996	5.9997	5.9996
	(H(4))	7.9994	7.9992	7.9994	7.9993	7.9993	7.9993	7.9993	7.9992	7.9994	7.9992
	(H(5))	9.9981	9.9979	9.9980	9.9979	9.9981	9.9979	9.9980	9.9977	9.9980	9.9979
	(H(6))	11.9925	11.9923	11.9924	11.9922	11.9924	11.9921	11.9924	11.9920	11.9925	11.9925
	Bit p(1)	0.5000	0.5004	0.5001	0.5003	0.4993	0.5001	0.4997	0.4994	0.4999	0.5003
	zlib ratio	0.1498	0.1499	0.1499	0.1498	0.1498	0.1499	0.1498	0.1498	0.1499	0.1498

## Appendix G

**Table A8:** Summary of normalized rule entropy and dominant-rule ratio for the eight DNA-to-bit coding rules under the R1, R1-ext, R2, and R3 settings.

Regime	Normalized Rule Entropy, Mean ± SD	Mean Dominant-Rule Ratio	Interpretation
R1	0.9999965 ± 0.0000015	0.1257	Rule selection did not collapse to a single rule
R1-ext	0.9999961 ± 0.0000011	0.1258	Rule usage remained balanced under the independent genomic source
R2	0.9999960 ± 0.0000016	0.1258	Rule usage remained balanced under the synthetic-data setting
R3	0.9999977 ± 0.0000011	0.1256	Balanced rule usage was maintained without training

Note: Values were computed from the usage counts of the eight DNA-to-bit coding rules recorded across 10 independent runs for each setting. A normalized rule entropy close to 1 indicates balanced rule usage. The dominant-rule ratio denotes the proportion of the most frequently selected rule in each run. Run-level rule traces and raw outputs are provided in the GitHub repository for reproducibility.

## Appendix H

**Table A9:** Run-level results for the R3 component-sensitivity and failure-oriented stress control.

Variant	Seed	GC Ratio	DNA Entropy (bits/base)	Max Homopolymer	Bit p(1)	Monobit *p*-Value	Normalized Rule Entropy	Dominant Rule Ratio
fixed-rule	8687970821042739881	0.499868	1.999219	5	0.491490	5.84 × 10^−65^	0.0000000	1.000000
fixed-rule	2717860915976751978	0.498612	1.999043	5	0.489772	5.31 × 10^−93^	0.0000000	1.000000
fixed-rule	4168663315303670174	0.496648	1.999152	5	0.490102	3.22 × 10^−87^	0.0000000	1.000000
no-homopolymer-mask	8687970821042739881	0.493784	1.998620	20	0.500057	0.9092	0.9999964	0.125958
no-homopolymer-mask	2717860915976751978	0.492966	1.998405	16	0.499972	0.9553	0.9999993	0.125396
no-homopolymer-mask	4168663315303670174	0.491918	1.998283	18	0.499714	0.5673	0.9999988	0.125538
no-lag1-suppression	8687970821042739881	0.498424	1.999216	5	0.500021	0.9660	0.9999963	0.125972
no-lag1-suppression	2717860915976751978	0.497458	1.999053	5	0.500025	0.9601	0.9999994	0.125350
no-lag1-suppression	4168663315303670174	0.496718	1.999047	5	0.499911	0.8587	0.9999987	0.125520
no-constraint	8687970821042739881	0.490798	1.992620	19	0.500269	0.5906	0.9999979	0.125418
no-constraint	2717860915976751978	0.488500	1.992336	23	0.500611	0.2217	0.9999972	0.125792
no-constraint	4168663315303670174	0.488796	1.992115	20	0.499805	0.6968	0.9999989	0.125310

**Table A10:** Summary statistics for the R3 stress variants.

Variant	GC Ratio, Mean ± SD	DNA Entropy, Mean ± SD	Max Homopolymer, Mean ± SD	Bit p(1), Mean ± SD	Monobit *p*-Value, Mean ± SD	Normalized Rule Entropy, Mean ± SD	Dominant Rule Ratio, Mean ± SD
fixed-rule	0.498376 ± 0.001623	1.999138 ± 0.000089	5.00 ± 0.00	0.490455 ± 0.000912	1.95 × 10^−65^ ± 3.37 × 10^−65^	0.0000000 ± 0.0000000	1.000000 ± 0.000000
no-homopolymer-mask	0.492889 ± 0.000935	1.998436 ± 0.000171	18.00 ± 2.00	0.499914 ± 0.000179	0.8106 ± 0.2120	0.9999982 ± 0.0000016	0.125631 ± 0.000292
no-lag1-suppression	0.497533 ± 0.000855	1.999105 ± 0.000096	5.00 ± 0.00	0.499986 ± 0.000065	0.9283 ± 0.0605	0.9999981 ± 0.0000016	0.125614 ± 0.000321
no-constraint	0.489365 ± 0.001250	1.992357 ± 0.000254	20.67 ± 2.08	0.500228 ± 0.000405	0.5030 ± 0.2493	0.9999980 ± 0.0000009	0.125507 ± 0.000253
